# Heat and mass flux through a Reiner–Rivlin nanofluid flow past a spinning stretching disc: Cattaneo–Christov model

**DOI:** 10.1038/s41598-022-18609-7

**Published:** 2022-08-24

**Authors:** Galal M. Moatimid, Mona A. A. Mohamed, Khaled Elagamy

**Affiliations:** Department of Mathematics, Faculty of Education, Ain Shams University, Roxy, Cairo, Egypt

**Keywords:** Biophysics, Materials science, Mathematics and computing, Nanoscience and technology

## Abstract

The current work scrutinizes a non-Newtonian nanofluid free convective flow induced by a rotating stretchable disc. The examination surveys the Stefan blowing and Cattaneo–Christov mass and heat fluxes, as a precise illustrative model. The innovative aspects of the ongoing project include the analysis of the border sheet nanofluid flow near a revolving disc through thermophoresis, Reiner–Rivlin prototype features, and random nanoparticle motion. The Reiner–Rivlin non-Newtonian model is considered together with the effect of an unvarying axial magnetic strength. The constitutive formulae of a Reiner–Rivlin liquid have been reproduced in the cylindrical coordinates. Through implementing the applicable relationship transformations, the controlling partial differential equations are transferred to ordinary differential equations (ODE). This procedure yields a group of coupled nonlinear ordinary differential equations in relation to speed, heat, and nanoparticle concentration profiles that are impacted by several physical characteristics. These equations are analyzed by using the homotopy perturbation method (HPM). Due to the analytical solution given by HPM, the current work enables us to take the infinity of the layer as a parameter of the problem and discuss its variation in the obtained distributions. Consequently, a physical significant graphical visualization of the data is emphasized. The rates of mass and temperature transmission are examined to understand if any of the relevant parameters may improve these rates. Additionally, the Stefan blowing causes extra particles diffusion, which enhances heat transfer and raises the nanoparticles concentration and could be useful in some medical therapies. Furthermore, the stretching of the rotating disc is concluded, which improves the fluid heat transfer.

## Introduction

The innovative potential in several biological sciences, biomedical (medicine) technological aspects, and non-Newtonian are the (Reiner–Rivlin) nanofluid mechanisms. Additionally, a variety of biological liquids including blood, synovial fluid, and saliva, exhibits a non-Newtonian behavior, and significant viscoelastic properties are demonstrated. Hayat et al.^1^ investigated the hydromagnetic movement of a Reiner–Rivlin nano liquid over a revolving disc. In their work, the physical aspect of entropy optimization was addressed. Numerical analysis was done on the thermodynamic characteristics of the Reiner–Rivlin nano liquid movement caused by a rotating disc^2^. To examine how nanoparticles affect the thermodynamics of the Reiner–Rivlin nanomaterial, the non-homogeneous two-phase nano-liquid model was studied. Numerous limiting instances were reported to be in strong accord with the bench marking investigations^3^. The drag force and the normalized force of a spherical bubble comprising a Reiner–Rivlin flow of a sheet of a surfactant film flowing in a Newtonian fluid were estimated^4^. The results of this framework can be employed in a wide range of engineering settings, in addition to chemical, biochemical, and environmental disciplines. Using the cell model technique, Jaiswal and Gupta^5^ provided an examination of viscous incompressible liquid Stokes flowing through a swarm of immiscible Reiner–Rivlin fluid droplets in a cell. A Reiner–Rivlin viscoelastic flowing incorporating Cattaneo–Christov (CC) temperature distribution in a permeable material across a roughly rotating disc was examined^3^. Concentration and temperature profiles were improved as a result of Reiner–Rivlin material parameters. He et al.^6^ looked at a planar boundary separating two bounded horizontal magnetic liquids, with the act of a uniform longitudinal magnetic strength. The insinuation of the linear as well as nonlinear curves showed that the linear curve governs the stability zones. A well-known approximation for a viscous flow between two flat plates, one of which rotates, and the other is stationary, was taken into consideration^7^. The topic has drawn considerable interest, particularly for Navier–Stokes flow**.** Nevertheless, the description of a relatively non-Newtonian fluid as elucidated by Reiner–Rivlin theory seems to be of more significance. Practically speaking, organized convective cooling technologies involve a coolant imposed on a flat plate and can use these fluxes. This phenomenon also happens frequently in a wide variety of technical and manufacturing uses, including treating wastewater, revolving machinery, medical instruments, spinning blades, computer storage systems, etc. Additionally, all rounded spinning bodies moving in a fluid experience a forced flow caused by a rotating disc, hence the current analysis satisfies the indicated requirements to assess the precise analytical solution to real-world issues. Therefore, the goal of the current investigation is to broaden this research to non-Newtonian cases**,** while taking the Reiner–Rivlin fluid model into consideration.

Nanofluids are fluids, including nanoparticles**,** which demonstrate considerable thermal improvement at low volume fractions. Many of the works on nanofluids aim to gain a better knowledge of their behavior in such a way as to employ them in such fields where heat transfer enhancement is critical, such as nuclear reactors, transport, technology, pharmacology, and foodstuffs**,** to mention but a few. It has also been suggested that nanofluid**s** can be used as a smart liquid, permitting heat transmission to be controlled or increase suddenly. Wong and De Leon^8^ emphasized that nanofluids have a wide variety of present and prospective applicability, stressing their improved heat transfer capabilities that are predictable, and their unique characteristics that make them convenient for such requirements. In a few words, nanofluids are liquid concentrations of nanomaterials having a fundamental dimension of fewer than 100 nm. An investigation of the stagnation point flow of a Cu-water nanofluid across an extending and contracting layer was conducted^9^. The case when there is a delay before feeling the beginning of disruptions across the material is the subject of this study. It was claimed that meteorological and environmental research, biochemical engineering, the generation of power and mobility, the translation of solar energy, electronic properties, sensor microfluidics, tumbler in polymer manufacture, and other sectors might benefit from this phenomenon^10^. A mathematical framework of an MHD mixed convective Cu-water nanofluid border layer movement across a surface stretched surface was examined^11^. The innovative feature of this project is the use of a modified Buongiorno type to account for Brownian motion, thermophoresis, and volume fraction for nanofluids. An attempt was undertaken to theoretically explore the influence of volume fraction on a mixed convective Cu-water nanofluid flow across a stretching surface with activation energy and thermal radiation, due to the growing demand for regulated cooling systems^12^. The influences of mixed convection, thermal radiation, and chemical reaction were numerically investigated for the steady Cu-water nanofluid flow in the presence of a magnetic field^13^. Heat transmission in a tube with non-parallel sides was analyzed^14^. Cu and Ag nanoparticles were used as the base fluid, and water was utilized as a solvent. The thermocapillary flow qualities and heat exchange efficiency of nanofluid droplets on a range of surfaces were examined in order to identify the effects of the underlying structure of the nanofluid evaporating droplets^15^. Kumar et al.^16^ used hybrid nanofluids to investigate the stability of heat transfer caused by large characteristics as well as the variation of fluids under the influence of thermal radiation. According to the graphical data, large amounts of the radiation factor result in more heat transfer, meanwhile, greater values of the Prandtl number result in heat transfer suppression. An overview of several nanofluid instability concerns as a function of different hydrodynamic and hydromagnetic characteristics was provided^17^. Nanofluid investigations are gaining more attention from scientists in recent days due to their multiple applications that make it possible for many industrial heat transfer processes. The field of medicine also uses nanofluids, some of which contain nanodrugs. Additionally, they are used in hard drives, heating and cooling equipment, jet engines, turbine systems, optical sensors, and hard discs. As a result, the current work is compatible with these fluids.

The Cattaneo–Christov (CC) model successfully modified the Fourier model to include the crucial component of thermal and mass relaxation times. This demonstrates how a hyperbolic energy equation is created for a temperature field, allowing for the conduction of heat at a constrained velocity through the use of heat waves. Interesting real-world uses of this kind of heat transmission include nanofluidic flows and skin burns models. The CC with a varying entropy production and thermal relaxation time was studied^18^. The micropolar fluid that absorbs heat in the presence of, mixed convection and partial slip was examined. To examine the feature of heat and mass transfer, two different nanoparticles, a single-wall carbon nanotube and a multi-wall carbon nanotube, were taken into consideration. Using the effect of instigation energy with CC, an angled cylinder was inspected to investigate the stagnation place of the flowing of an organic molecule Powell–Eyring nanofluid^19^. The flow data was designed using the Buongiorno nanofluid framework, which took into consideration such nonlinear effects as infrared energy, binary chemical processes, and non-Fourier heat transfer. The movement, temperature distribution, and entropy formation of a non-Newtonian hybrid nanofluid through a lubricated surface with the concept of CC heat flux have not been yet studied, according to a thorough survey of the bibliography. The Casson fluid framework of the hybrid nanofluid through the lubricated surface with thermal radiation and viscous dissipation was therefore evaluated in a previous study^20^. The impact of the chemical reaction of the upper-connected Maxwell liquid was the focus of Khan et al.^21^. In such transmission, entropy minimizing in a hybrid nanofluid stagnation point flow across a nonlinearly extending layer in the presence of Thomson and Troian border requirements was considered^22^. The Darcy–Forchheimer relationship was established as a result of the porous medium. Using the CC heat flux framework, the nonlinear thermal propagation, heat production, and viscous dissipation were addressed. Heat and mass transfer processes were investigated using the CC methodology. Using mathematical formalism, Raju et al.^23^ explored the heat movement transmission parameters of a Maxwell liquid over a stretched surface with CC, heat source or sink, and suction/injection were surveyed. Subbarayudu et al.^24^ used initiating energies with a twofold chemical process across a stretched sheet to construct a conceptual model to examine the hydro-magnetic movement of carbon nanotubes immersed in a Maxwell nanofluid. Heat transfer processes in energy expression were investigated using both the dynamic absorption and nonlinear heat flux models through an original approach, as well as the CC prototype of temperature diffusion, which is a more advanced version than Fourier’s heat flux formula. Shankar et al.^25^ expounded the magnetic effects of CC double diffusion patterns on heat and mass transmission behavior of a viscous incompressible, time-dependent, two-dimensional Casson nanofluid movement across the channel with Joule heating and viscous dissipation impacts. The validity of the findings and the approaches employed to accomplish the goal of the present work was clearly demonstrated by a comparison between the results provided and the findings of earlier research. In light of the advantages of employing the CC model, the current problem is performed along with this approach.

The occurrence of ODEs/PDEs plays an essential role in several zones of science, physics, engineering, and ecology. The physical significance of the dynamical behaviors requires understanding the ODEs/PDEs. Many scientists have investigated nonlinear phenomena significantly in recent decades. In engineering implementations, the nonlinear behavior is more frequent than the linear one. Although linear systems can yield analytical solutions simply, only limited particular nonlinear schemes have analytical solutions. The perturbation theory is the first approach to yield approximate analytical nonlinear system solutions. As a new methodology of the perturbation theories, He^26^ is accredited as being the first to solve an ODE by introducing a synthetic incorporated parameter $q \in \left[ {0,\;1} \right]$ into the differential equation. Along with this parameter, the problem may be divided into two parts. Consequently, the artificial parameter divides the previous two parts. Therefore, He^26–29^ proposed a prospective and effective analytical method in this approach, which is abbreviated as (HPM). This technique is used to provide an analytical approximate solution for a variety of ODEs encountered in science and mathematics. When the HPM is compared to other perturbation methods, it is clear that it produces more accurate results. Therefore, the HPM has all of the advantages of the classical perturbation approaches**,** with no need for small parameter assumptions in the solution procedure. The approach has a simpler structure since it needs less calculation time and has rapid computational accuracy than the preceding classical techniques. To solve any problem, this method just needs primary circumstances in such a way as to produce an analytical approximate solution as an infinite power series. HPM is often used to solve NODEs by a number of researchers whose findings are easily presented. One of them is referred to as the linear part which must have an exact solution, whereas the other is known as the nonlinear part. The nonlinear EHD stability of a cylinder-shaped interface between two Walters' B-type viscoelastic liquids was examined in a permeable media^30^. The extended frequency theory was merged with the HPM to produce a distribution of the surface displacement in an analytical periodical approximation. A motile microbe behaving in an MHD movement of an incompressible nano liquid that followed the non-Newtonian Jeffrey prototype was investigated^31^. Using HPM, the fundamental equations of motion are analytically resolved. The non-Newtonian flow is followed by an incompressible nanofluid flow. The Casson prototype was employed for its non-Newtonian fluid behavior^32^. The flow fills up the conical space among the cone stationary, the revolving surfaces, and the horizontal disc. The HPM was used to analytically solve these equations. Owing to the highly nonlinear fundamental equations in the present study, the effective HPM is used to analyze the problem at hand. In a conclusion, the HPM can be used to investigate a wide range of additional nonlinear issues of scientific and technological significance, especially in linear and nonlinear wave mechanical and vibrational issues.

The current study investigates the Reiner–Rivlin fluid model induced by a rotating stretchy disc with Stefan blowing and the CC modeling, in addition to the above-mentioned characteristics. It is considered that there is a constant magnetic field. The controlling equations of motion are converted into a new group of ODEs using appropriate similarity modifications. Food manufacturing, heat transfer absorption throughout paper drying operations, and separating processes are affected by the main challenge of the present paper.

The answers to the following relevant queries are anticipated at the conclusion of this investigation:

- What are the mechanisms of heat and mass transmission through nanofluids under the effects of Stefan blowing and the CC model?
- Does the Reiner–Rivlin model cause major deviations in the thermal boundary of a rotating disc?
- Does the expanding nature of the revolving disc enhance the flow, heat, and nanoparticle transfer rates?
- What are the effects of thermophoresis and Brownian motion features on the flow?

The remainder of the paper is structured as follows: The approach taken to this issue is outlined in section “Problem structure”. The essential boundary conditions are included in this section together with the governing equations of motion. In section “Straightforward changes of correlation”, the boundary-value problem is covered utilizing simple similarity transformations. How to use the HPM to develop the analytical solution is described in section “Methodology of solution”. The conclusions and comments are introduced in section “Results and discussion”. The main findings are subsequently reported as closing remarks in section “Conclusions”.

## Problem structure

**Figure 1:**
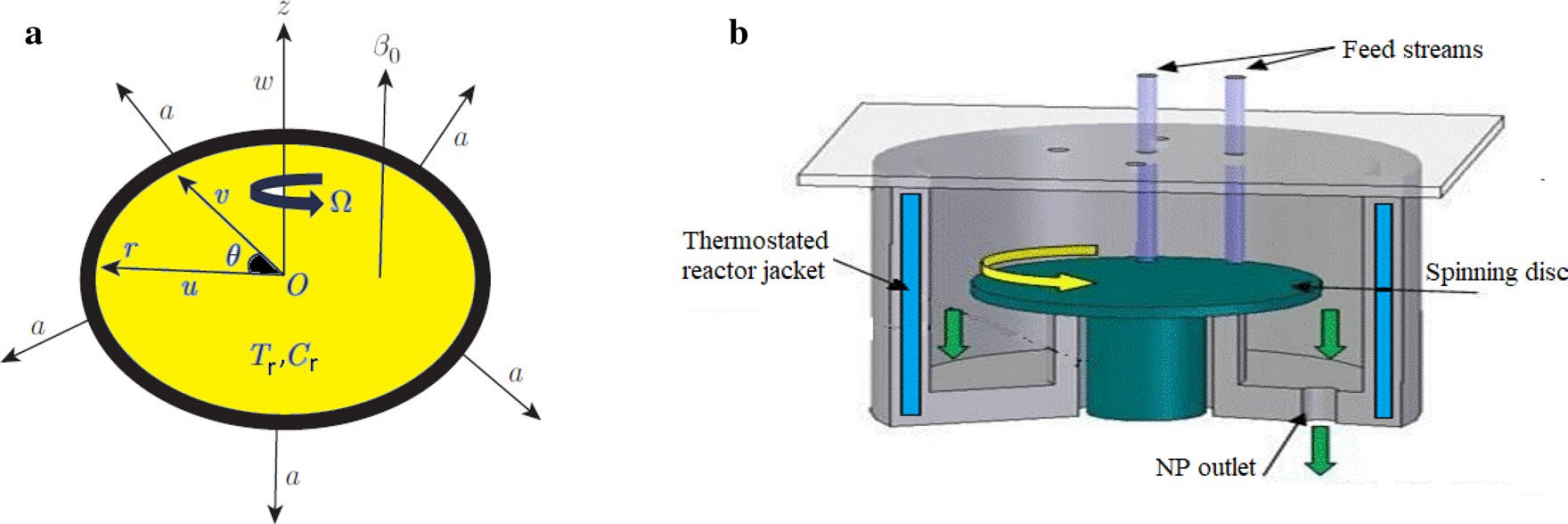
(**a**) Physical flow configuration. (**b**) A design of the spinning disc reactor (SDR).

The configuration addresses a two-dimensional MHD non-Newtonian nanofluid obeying a Reiner–Rivlin representation, flowing past a rotating stretching disc. The model utilizes cylindrical coordinates $(r,\;\theta ,\;z)$ for ease of use. The disc is located at $z = 0$ and spins with a uniform angular velocity $\Omega$ across the axial direction, while it stretches in the radial path with a stable stretched parameter *a*. It is planned to provide a magnetic field $B_{0}$ parallel to the *z*-axis that is strong and consistent. The revolving surface is excited by a warmer fluid of temperature *T*_*r*_ and meditation* C*_*r*_, whilst in free stream; the surrounding fluid is kept at constant values of temperature $T_{\infty }$ and nanoparticles $C_{\infty }$. Additionally, along with the normal velocity condition, the flat surface is disposed to border circumstances, having mass concentration with the Stefan blowing effect. In accordance with the above-mentioned construction, the configuration model is clarified and shown in Fig. 1a.

The spinning disc reactor (SDR), shown in Fig. 1b, is one of the most significant applications of our model. Producing nanoparticles with regulated qualities, such as particle size and distribution, is easy with this equipment. Over the past few decades, this technique has been successfully established. The velocity of the disc, its surface type (smooth or grooved), the feed liquid flow rates, the number of feeding points and their positions, and the presumptive stabilization manager are the main SDR control strictures. The liquid combinations are concentrated in the disc center and rotate at high speeds (between 300 and 3000 rpm), forming a thin fluid layer (1–200 nm). Large heat and mass transmission is induced by the fluid layer thickness and the wide contact area it has with the disc surface. Finally, highly quick and efficient mixing (micro-mixing) of the components of the liquid flowing over the disc surface is made possible by the drag forces between the fluid flowing and the disc surface^33^.

### Controlling equations of motion

The Cauchy stress tensor $\underline{\underline{\tau }}$ is used for the Reiner–Rivlin fluid^34^ and is defined as follows:

1 $$\underline{\underline{\tau }} = - P\underline{\underline{I}} + \mu_{f} \underline{\underline{A}} + \mu_{c} \underline{\underline{A}}^{2} ,$$

where $\underline{\underline{A}} = \nabla \underline{V} + \nabla \underline{V}^{T}$ is the first order Rivlin–Ericksen tensor, $\underline{V}$ represents the fluid velocity and $\underline{\underline{I}}$ is the identity tensor.

The conservation of mass and momentum equations of an incompressible non-Newtonian liquid may be described as follows^2,35^:

2 $$\nabla \cdot \underline{V} = 0,$$

and

3 $$\rho_{f} \left( {\frac{{\partial \underline{V} }}{\partial t} + \underline{V} \cdot \nabla \underline{V} } \right) = \nabla \cdot \underline{\underline{\tau }} + \underline{J} \times \underline{B} ,$$

where $\rho_{f}$ is the fluid density, $\underline{J}$ is the current density and $\underline{B}$ is the magnetic strength.

The temperature and nanoparticles dissipation equations^25,36^ of fluid may be written as:

4 $$(\rho c)_{f} \left( {\frac{\partial T}{{\partial t}} + \underline{V} \cdot \nabla T} \right) = - \nabla \cdot \underline{Q} ,$$

and

5 $$\frac{\partial C}{{\partial t}} + \underline{V} \cdot \nabla C = - \nabla \cdot\underline{j} ,$$

where $T$ is the fluid heat, $\underline{Q}$ is the thermal flux, $C$ is nanoparticles concentration and $\underline{j}$ is the mass flux. The CC diffusion theory^37,38^ is employed to analyse the heat and mass flow performance of nano-fluids instead of the conventional transport models. To investigate this impact, the upper-convective material time derivative of a vector $\underline{F}$ has the form:

6 $$\frac{{d\underline{F} }}{dt} = \frac{{\partial \underline{F} }}{\partial t} + \underline{V} \cdot\nabla \underline{F} + (\nabla \cdot\underline{V} )\underline{F} - \underline{F} \cdot\nabla \underline{V} .$$

In terms of CC representation, the Fourier’s law for both the thermal flux $\underline{Q}$ and the mass change $\underline{j}$ have been generalized as follows:

7 $$\, \underline{Q} + \, \lambda_{E} \left( {\frac{{\partial \underline{Q} }}{\partial t} + \underline{V} \cdot\nabla \underline{Q} + (\nabla \cdot\underline{V} )\underline{Q} - \underline{Q} \cdot\nabla \underline{V} } \right) = - \alpha_{m} \nabla T,$$

8 $$\underline{j} + \lambda_{C} \left( {\frac{{\partial \underline{j} }}{\partial t} + \underline{V} \cdot\nabla \underline{j} + (\nabla \cdot\underline{V} )\underline{j} - \underline{j} \cdot\nabla \underline{V} } \right) = - D_{B} \nabla C,$$

where $\lambda_{E}$ is the heat flux relaxation time, $\alpha_{m}$ is the thermal conductivity, $\lambda_{C}$ is the mass flux relaxation time, and $D_{B}$ is the Brownian diffusivity factor. On the other hand, the standard Fourier and Fick laws may be required in return by putting the coefficients $\lambda_{E} = \lambda_{C} = 0$ in the above equations, correspondingly.

Utilizing the situation of incompressibility $\nabla \cdot\underline{V} = 0$ and the situation of stationary flow that is $\frac{{\partial \underline{Q} }}{\partial t} = 0$ and $\frac{{\partial \underline{j} }}{\partial t} = 0$, the Eqs. (7) and (8) change over to

9 $$\, \underline{Q} = - \alpha_{m} \nabla T - \lambda_{E} \left( {\underline{V} \cdot\nabla \underline{Q} - \underline{Q} \cdot\nabla \underline{V} } \right),$$

10 $$\underline{j} = - D_{B} \nabla C - \lambda_{C} \left( {\underline{V} \cdot\nabla \underline{j} - \underline{j} \cdot\nabla \underline{V} } \right).$$

By reducing $\underline{Q}$ and $\underline{j}$ in Eqs. (4) and (5), and using Eqs. (9) and (10), the heat and nanoparticles equations with the Brownian and thermophoresis properties become

11 $$\underline{V} \cdot\nabla T + \lambda_{E} \Phi_{E} = \frac{{\alpha_{m} }}{{(\rho c)_{f} }}\left( {\nabla^{2} T} \right) + \frac{{(\rho c)_{p} }}{{(\rho c)_{f} }}\left( {D_{B} \left( {\nabla T\cdot\nabla C} \right) + \frac{{D_{T} }}{{T_{\infty } }}\left( {\nabla T} \right)^{2} } \right),$$

and

12 $$\underline{V} \cdot\nabla T + \lambda_{C} \Phi_{C} = D_{B} \left( {\nabla^{2} C} \right) + \frac{{D_{T} }}{{T_{\infty } }}\left( {\nabla^{2} T} \right),$$

such that $\Phi_{E}$ and $\Phi_{C}$ are the modified expressions for CC theory. In light of the above-mentioned description, these expressions^35^ are defined as follows:

13 $$\Phi_{E} = u\frac{\partial T}{{\partial r}}\frac{\partial u}{{\partial r}} + w\frac{\partial T}{{\partial r}}\frac{\partial u}{{\partial z}} + u\frac{\partial T}{{\partial z}}\frac{\partial w}{{\partial r}} + w\frac{\partial T}{{\partial z}}\frac{\partial w}{{\partial z}} + 2uw\frac{{\partial^{2} T}}{\partial r\partial z} + w^{2} \frac{{\partial^{2} T}}{{\partial z^{2} }} + u^{2} \frac{{\partial^{2} T}}{{\partial r^{2} }},$$

14 $$\Phi_{C} = u\frac{\partial C}{{\partial r}}\frac{\partial u}{{\partial r}} + w\frac{\partial C}{{\partial r}}\frac{\partial u}{{\partial z}} + u\frac{\partial C}{{\partial z}}\frac{\partial w}{{\partial r}} + w\frac{\partial C}{{\partial z}}\frac{\partial w}{{\partial z}} + 2uw\frac{{\partial^{2} C}}{\partial r\partial z} + w^{2} \frac{{\partial^{2} C}}{{\partial z^{2} }} + u^{2} \frac{{\partial^{2} C}}{{\partial r^{2} }},$$

Simultaneously, the governing equations of motion concerning continuity and momentum of the Reiner–Rivlin fluid may be formulated as follows:

15 $$\frac{\partial u}{{\partial r}} + \frac{u}{r} + \frac{\partial w}{{\partial z}} = 0,$$

16 $$\rho_{f} \left( {u\frac{\partial u}{{\partial r}} + w\frac{\partial u}{{\partial z}} - \frac{{v^{2} }}{r}} \right) = \frac{{\partial \tau_{rr} }}{\partial r} + \frac{{\partial \tau_{zr} }}{\partial z} + \frac{{\tau_{rr} - \tau_{\theta \theta } }}{r} - \sigma B_{o}^{2} u,$$

17 $$\rho_{f} \left( {u\frac{\partial v}{{\partial r}} + w\frac{\partial v}{{\partial z}} + \frac{uv}{r}} \right) = \frac{1}{{r^{2} }}\frac{{\partial (r^{2} \tau_{r\theta } )}}{\partial r} + \frac{{\partial \tau_{z\theta } }}{\partial z} - \sigma B_{o}^{2} v,$$

18 $$\rho_{f} \left( {u\frac{\partial w}{{\partial r}} + w\frac{\partial w}{{\partial z}}} \right) = \frac{1}{r}\frac{{\partial (r\tau_{rz} )}}{\partial r} + \frac{{\partial \tau_{zz} }}{\partial z},$$

where the mathematical expression of the components $\tau_{ij}$ of the Reiner–Rivlin fluid may be listed as follows:

19 $$\tau_{rr} = - P + 2\mu_{f} \left( {\frac{\partial u}{{\partial r}}} \right) + \mu_{c} \left[ {4\left( {\frac{\partial u}{{\partial r}}} \right)^{2} + \left( {\frac{\partial v}{{\partial r}} - \frac{v}{r}} \right)^{2} + \left( {\frac{\partial u}{{\partial z}} + \frac{\partial w}{{\partial r}}} \right)^{2} } \right],$$

20 $$\tau_{zr} = \mu_{f} \left( {\frac{\partial u}{{\partial z}} + \frac{\partial w}{{\partial r}}} \right) + \mu_{c} \left[ {2\left( {\frac{\partial u}{{\partial r}}} \right)\left( {\frac{\partial u}{{\partial z}} + \frac{\partial w}{{\partial r}}} \right) + \left( {\frac{\partial v}{{\partial r}} - \frac{v}{r}} \right)\left( {\frac{\partial v}{{\partial z}}} \right) + \left( {\frac{\partial u}{{\partial z}} + \frac{\partial w}{{\partial r}}} \right)\left( {2\frac{\partial w}{{\partial z}}} \right)} \right],$$

21 $$\tau_{\theta \theta } = - P + \mu_{f} \left( \frac{2u}{r} \right) + \mu_{c} \left[ {4\left( \frac{u}{r} \right)^{2} + \left( {\frac{\partial v}{{\partial r}} - \frac{v}{r}} \right)^{2} + \left( {\frac{\partial v}{{\partial z}}} \right)^{2} } \right],$$

22 $$\tau_{r\theta } = \mu_{f} \left( {\frac{\partial v}{{\partial r}} - \frac{v}{r}} \right) + \mu_{c} \left[ {2\left( {\frac{\partial u}{{\partial r}}} \right)\left( {\frac{\partial v}{{\partial r}} - \frac{v}{r}} \right) + \left( \frac{2u}{r} \right)\left( {\frac{\partial v}{{\partial r}} - \frac{v}{r}} \right) + \left( {\frac{\partial u}{{\partial z}} + \frac{\partial w}{{\partial r}}} \right)\left( {\frac{\partial v}{{\partial z}}} \right)} \right],$$

23 $$\tau_{z\theta } = \mu_{f} \left( {\frac{\partial v}{{\partial z}}} \right) + \mu_{c} \left[ {\left( {\frac{\partial u}{{\partial z}} + \frac{\partial w}{{\partial r}}} \right)\left( {\frac{\partial v}{{\partial r}} - \frac{v}{r}} \right) + \left( \frac{2u}{r} \right)\left( {\frac{\partial v}{{\partial z}}} \right) + 2\left( {\frac{\partial w}{{\partial z}}} \right)\left( {\frac{\partial v}{{\partial z}}} \right)} \right],$$

and

24 $$\tau_{zz} = - P + 2\mu_{f} \left( {\frac{\partial w}{{\partial z}}} \right) + \mu_{c} \left[ {\left( {\frac{\partial v}{{\partial z}}} \right)^{2} + \left( {\frac{\partial u}{{\partial z}} + \frac{\partial w}{{\partial r}}} \right)^{2} + 4\left( {\frac{\partial w}{{\partial z}}} \right)^{2} } \right].$$

The temperature equation then becomes

25 $$\left. \begin{aligned} &u\frac{\partial T}{{\partial r}} + w\frac{\partial T}{{\partial z}} + \lambda_{E} \left( {u\frac{\partial T}{{\partial r}}\frac{\partial u}{{\partial r}} + w\frac{\partial T}{{\partial r}}\frac{\partial u}{{\partial z}} + u\frac{\partial T}{{\partial z}}\frac{\partial w}{{\partial r}} + w\frac{\partial T}{{\partial z}}\frac{\partial w}{{\partial z}} + 2uw\frac{{\partial^{2} T}}{\partial r\partial z} + w^{2} \frac{{\partial^{2} T}}{{\partial z^{2} }} + u^{2} \frac{{\partial^{2} T}}{{\partial r^{2} }}} \right) \hfill \\ &\quad = \frac{{\alpha_{m} }}{{(\rho c)_{f} }}\left( {\frac{{\partial^{2} T}}{{\partial r^{2} }} + \frac{1}{r}\frac{\partial T}{{\partial r}} + \frac{{\partial^{2} T}}{{\partial z^{2} }}} \right) + \frac{{(\rho c)_{p} }}{{(\rho c)_{f} }}\left( {D_{B} \left( {\frac{\partial T}{{\partial r}}\frac{\partial C}{{\partial r}} + \frac{\partial T}{{\partial z}}\frac{\partial C}{{\partial z}}} \right) + \frac{{D_{T} }}{{T_{\infty } }}\left( {\left( {\frac{\partial T}{{\partial r}}} \right)^{2} + \left( {\frac{\partial T}{{\partial z}}} \right)^{2} } \right)} \right) \hfill \\ \end{aligned} \right\}.$$

Additionally, the nanoparticles concentration equation gives

26 $$\left. \begin{aligned} &u\frac{\partial C}{{\partial r}} + w\frac{\partial C}{{\partial z}} + \lambda_{C} \left( {u\frac{\partial C}{{\partial r}}\frac{\partial u}{{\partial r}} + w\frac{\partial C}{{\partial r}}\frac{\partial u}{{\partial z}} + u\frac{\partial C}{{\partial z}}\frac{\partial w}{{\partial r}} + w\frac{\partial C}{{\partial z}}\frac{\partial w}{{\partial z}} + 2uw\frac{{\partial^{2} C}}{\partial r\partial z} + w^{2} \frac{{\partial^{2} C}}{{\partial z^{2} }} + u^{2} \frac{{\partial^{2} C}}{{\partial r^{2} }}} \right) \\ &\quad = D_{B} \left( {\frac{{\partial^{2} C}}{{\partial r^{2} }} + \frac{1}{r}\frac{\partial C}{{\partial r}} + \frac{{\partial^{2} C}}{{\partial z^{2} }}} \right) + \frac{{D_{T} }}{{T_{\infty } }}\left( {\frac{{\partial^{2} T}}{{\partial r^{2} }} + \frac{1}{r}\frac{\partial T}{{\partial r}} + \frac{{\partial^{2} T}}{{\partial z^{2} }}} \right) \hfill \\ \end{aligned} \right\}.$$

The aforementioned main equations of motion should be valid to the suitable border situations. The suitable border situations were given by^35^. These circumstances may be listed as follows:

27 $$\left. {\begin{array}{*{20}c} {u = ar,\;v = r\Omega ,\;w = - \frac{{D_{B} }}{{1 - C_{r} }}\left( {\frac{\partial C}{{\partial z}}} \right),\;T = T_{r} + \beta_{1} \frac{\partial T}{{\partial z}},\;C = C_{r} + \beta_{2} \frac{\partial C}{{\partial z}}\quad at\quad z = 0} \\ {u \to 0,\;v \to 0,\;T \to T_{\infty } ,\;C \to C_{\infty } \quad as\quad z \to \infty } \\ \end{array} } \right\},$$

where *β*_1_ and *β*_2_ are the slip coefficients of heat and mass, respectively. Now, the boundary-value problem of this manuscript becomes well-defined. The method of solution will be outlined in section “Methodology of solution”.

### Supplementary amounts of attention

The skin friction factor $C_{f}$, the Nusselt numeral $Nu$ and the Sherwood numeral $Sh$ are among the most important physical amounts that are related to the topic. These amounts are mathematically well-described as:

28 $$C_{f} = \left. {\sqrt {\tau_{r}^{2} + \tau_{\theta }^{2} } } \right|_{z = 0} /\rho_{f} (r\Omega )^{2} ,$$

29 $$Nu = - r\alpha_{m} \left. {\frac{\partial T}{{\partial z}}} \right|_{z = 0} /\alpha_{m} (T_{r} - T_{\infty } ),$$

and

30 $$Sh = - rD_{B} \left. {\frac{\partial C}{{\partial z}}} \right|_{z = 0} /D_{B} (C_{r} - C_{\infty } ).$$

From a physical standpoint:

- The skin friction factor $C_{f}$ is produced by the viscosity of fluids. It has been recognized as the laminar drag in the turbulent drag. Skin friction drag is usually connected to families of the Reynolds numeral, which is the relationship between the inertial force and the viscous force.
- The Nusselt number $Nu$ is the proportion between convection and thermal conduction over a boundary.
- Finally, the Sherwood number $Sh$ represents the proportion between mass transmission by convection and mass transfer by dispersion.

## Straightforward changes of correlation

The foremost nonlinear partial equations are simply converted into ODEs through an appropriate similarity conversion. In view of Refs.^2,35^, the appropriate similarity transformations may be expressed as:

31 $$\left. \begin{gathered} u = \Omega rf^{\prime}(\eta ),\,\;v = \Omega rg(\eta ),\,\;w = - 2\sqrt {\Omega \upsilon } f(\eta ),\;P = \rho \Omega \nu P(\eta ), \hfill \\ \theta (\eta ) = \frac{{T - T_{\infty } }}{{T_{r} - T_{\infty } }},\,\;\varphi (\eta ) = \frac{{C - C_{\infty } }}{{C_{r} - C_{\infty } }},\,\,\quad {\text{and}}\,\,\quad \eta = \sqrt {\frac{\Omega }{\nu }} \,z, \hfill \\ \end{gathered} \right\},$$

where the dash signifies the differentiation with reference to the independent parameter $\eta$.

Inserting these transformations into the aforementioned governing equations of motion, one finds

32 $$f^{\prime\prime\prime} - f^{{\prime}{2}} + 2ff^{\prime\prime} + g^{2} - K(2f^{\prime}f^{\prime\prime\prime} - f^{\prime\prime} + g^{{\prime}{2}} ) - M^{2} f^{\prime} = 0,$$

33 $$g^{\prime\prime} + 2K(f^{\prime\prime}g^{\prime} - f^{\prime}g^{\prime}) - 2f^{\prime}g + 2fg^{\prime} - M^{2} g = 0,$$

34 $$\theta ^{\prime\prime} + 2{\text{Pr }} f\theta ^{\prime} - 4{\text{Pr }} \lambda_{1} (ff^{\prime}\theta ^{\prime} + f^{2} \theta ^{\prime\prime}) + {\text{Pr }} N_{B} \theta ^{\prime}\varphi ^{\prime} + {\text{Pr }} N_{T} \theta ^{{\prime}{2}} = 0,$$

35 $$\varphi ^{\prime\prime} + \frac{{N_{T} }}{{N_{B} }}\theta ^{\prime\prime} + 2S_{c} f\varphi ^{\prime} - 4\lambda_{2} S_{c} (ff^{\prime}\varphi ^{\prime} + f^{2} \varphi ^{\prime\prime}) = 0,$$

36 $$C_{f} = {\text{Re}}^{ - 1/2} \,\sqrt {f^{{\prime\prime}{2}} (0) + g^{{\prime}{2}} (0)} ,$$

37 $$Nu = {\text{Re}}^{1/2} \,\theta ^{\prime}(0),$$

and

38 $$Sh = {\text{Re}}^{1/2} \,\varphi ^{\prime}(0),$$

where ${\text{Re}} = \sqrt {\Omega /\upsilon \,} r$ is the Reynolds numeral.

Moreover, the border restrictions as given in Eq. (27) have been transformed to:

39 $$\left. {\begin{array}{*{20}c} {f^{\prime}(0) = \alpha ,\;g(0) = 1,\;f(0) = \frac{{S_{w} }}{{S_{c} }}\varphi ^{\prime}(0),\;\theta (0) = 1 + \gamma \,\theta ^{\prime}(0),\;\varphi (0) = 1 + \delta \,\varphi ^{\prime}(0)} \\ {f^{\prime} \to 0,\;g \to 0,\;\theta \to 0,\;\varphi \to 0\quad as\quad \eta \to \eta_{\infty } } \\ \end{array} } \right\},$$

where, $\eta_{\infty }$ represents the infinity value.

The significant non-dimensional parameters concluded in Eqs. (32–38) are defined in Ref.^35^ as follows:

The physical restriction of the Reiner–Rivlin fluid is $K = \mu_{c} \Omega /\mu_{f}$, the magnetic factor is $M = \sigma B_{0}^{2} \upsilon /\Omega \mu_{f}$, the Prandtl numeral is ${\text{Pr}} = \upsilon (\rho c)_{f} /\alpha_{m}$, the Deborah numeral due to the energy dispersion is $\lambda_{1} = \Omega \lambda_{E}$, the Brownian motion parameter is $N_{B} = (\rho c)_{p} D_{B} (C_{r} - C_{\infty } )/(\rho c)_{f} \upsilon$, the thermophoretic factor is $N_{T} = (\rho c)_{p} D_{T} (T_{r} - T_{\infty } )/(\rho c)_{f} \upsilon T_{\infty }$, the Schmidt factor is $S_{c} = \upsilon /D_{B}$, the Deborah numeral due to the mass dispersion is $\lambda_{2} = \Omega \lambda_{C}$, the Stefan blowing parameter is $S_{w} = ( {C_{r} - C_{\infty } } )/2(1 - C_{r} )$, the stretching parameter is $\alpha = \Omega /a$, the thermal slip parameter is $\gamma = \beta_{1} \sqrt {\Omega /\upsilon }$**,** and the nanoparticle volume fraction slip parameter is $\delta = \beta_{2} \sqrt {\Omega /\upsilon }$.

## Methodology of solution

As known, the main aim of all perturbation methods is transforming the NDEs into normal equations. Because the HPM^39,40^ is significant, effective, and promising, it will be adopted to analyze the fundamental organization of NDEs given by Eqs. (32–35) along with the border restrictions (39). The differential equation will be divided into linear and nonlinear portions. The two branches are disconnected by the embedded limitation, specifically $q \in \left[ {0,\,\,1} \right]$ to construct what is recognized by the Homotopy equation. Therefore, the above-mentioned equations may be formulated as follows

40 $$H(f,P) = L_{1} (f) + q\left[ {2ff^{\prime\prime} + g^{2} - K\left( {2f^{\prime}f^{\prime\prime\prime} + g^{{\prime}{2}} - f^{\prime\prime}} \right) - f^{{\prime}{2}} } \right] = 0,$$

41 $$H(g,P) = L_{2} (g) + q\left[ {2K\left( {f^{\prime\prime}g^{\prime} - f^{\prime}g^{\prime\prime}} \right) - 2f^{\prime}g + 2fg^{\prime}} \right] = 0,$$

42 $$H(\theta ,P) = L_{3} (\theta ) + q\left[ {2{\text{Pr}} f\theta ^{\prime} - 4{\text{Pr}} \lambda_{1} f\,f^{\prime}\theta ^{\prime} + f^{2} \theta ^{\prime\prime} + {\text{Pr}} N_{B} \theta ^{\prime}\varphi ^{\prime} + {\text{Pr}} N_{T} \theta ^{{\prime}{2}} } \right] = 0,$$

and

43 $$H(\varphi ,P) = L_{4} (\varphi ) + q\left[ {(N_{T} /N_{B} )\,\theta ^{\prime\prime} + 2S_{c} f\,\varphi ^{\prime} - 4\lambda_{2} S_{c} \left( {ff^{\prime}\varphi ^{\prime} + f^{2} \varphi ^{\prime\prime}} \right)} \right] = 0,$$

where $L_{1} \equiv \frac{{d^{3} }}{{d\eta^{3} }} - M^{2} \frac{d}{d\eta }$, $L_{2} \equiv \frac{{d^{2} }}{{d\eta^{2} }} - M^{2}$, $L_{3} \equiv \frac{{d^{2} }}{{d\eta^{2} }}$ and $L_{4} \equiv \frac{{d^{2} }}{{d\eta^{2} }}$ are the linear operators.

According to the aforementioned technique, the dependent variable may be expanded as follows:

44 $$\chi (\eta ,\;q) = \chi_{0} (\eta ) + q\chi_{1} (\eta ) + q^{2} \chi_{2} (\eta ) + \cdots ,$$

where $\chi (\eta ,\;q)$ stands for any of the functions $f^{\prime},\,\,g,\,\,\theta$ and $\varphi$.

Replacing Eq. (44) into Eqs. (40)–(43), after explanation and comparison of the similar powers of *q*-terms, one gets:

### Zero-order system

45 $$\frac{{d^{3} f_{o} }}{{d\eta^{3} }} - M^{2} \frac{{df_{0} }}{d\eta } = 0,$$

46 $$\frac{{d^{2} g_{0} }}{{d\eta^{2} }} - M^{2} g_{0} = 0,$$

47 $$\frac{{d^{2} \theta_{0} }}{{d\eta^{2} }} = 0,$$

and

48 $$\frac{{d^{2} \varphi_{0} }}{{d\eta^{2} }} = 0,$$

with the suitable border circumstances given as:

49 $$\left. {\begin{array}{*{20}c} {f_{0} ^{\prime}(0) = \alpha ,\;g_{0} (0) = 1,\;f_{0} (0) = \frac{{S_{w} }}{{S_{c} }}\varphi_{0} ^{\prime}(0),\;\theta_{0} (0) = 1 + \gamma \,\theta_{0} ^{\prime}(0),\;\varphi_{0} (0) = 1 + \delta \,\varphi_{0} ^{\prime}(0)} \\ {f_{0} ^{\prime} \to 0,\;g_{0} \to 0,\;\theta_{0} \to 0,\;\varphi_{0} \to 0\quad as\quad \eta \to \eta_{\infty } } \\ \end{array} } \right\}.$$

### First-order system

50 $$\frac{{d^{3} f_{1} }}{{d\eta^{3} }} - M^{2} \frac{{df_{1} }}{d\eta } = - 2f_{0} f_{0} ^{\prime\prime} - g_{0}^{2} + K\left( {2f_{0} ^{\prime}f_{0} ^{\prime\prime\prime} + g_{0} ^{{\prime}{2}} - f_{0} ^{\prime\prime}} \right) + f_{0} ^{{\prime}{2}} ,$$

51 $$\frac{{d^{2} g_{1} }}{{d\eta^{2} }} - M^{2} g_{1} = - 2K\left( {f_{0} ^{\prime\prime}g_{0} ^{\prime} - f_{0} ^{\prime}g_{0} ^{\prime\prime}} \right) + 2f_{0} ^{\prime}g_{0} + 2f_{0} g_{0} ^{\prime},$$

52 $$\frac{{d^{2} \theta_{1} }}{{d\eta^{2} }} = - 2{\text{Pr}} f_{0} \theta_{0} ^{\prime} + 4{\text{Pr}} \lambda_{1} \left( {f_{0} f_{0} ^{\prime}\theta_{0} ^{\prime} + f_{0}^{2} \theta_{0} ^{\prime\prime}} \right) - {\text{Pr}} N_{B} \theta_{0} ^{\prime}\varphi_{0} ^{\prime} - {\text{Pr}} N_{T} \theta_{0} ^{{\prime}{2}} ,$$

and

53 $$\frac{{d^{2} \varphi_{1} }}{{d\eta^{2} }} = - \frac{{N_{T} }}{{N_{B} }}\theta_{0} ^{\prime\prime} - 2S_{c} f_{0} \varphi_{0} ^{\prime} + 4\lambda_{2} S_{c} \left( {f_{0} f_{0} ^{\prime}\varphi_{0} ^{\prime} + f_{0}^{2} \varphi_{0} ^{\prime\prime}} \right),$$

only with appropriate border circumstances, they are as follows:

54 $$\left. {\begin{array}{*{20}c} {f_{1} ^{\prime}(0) = 0,\;g_{1} (0) = 0,\;f_{1} (0) = \frac{{S_{w} }}{{S_{c} }}\varphi_{1} ^{\prime}(0),\;\theta_{1} (0) = \gamma \,\theta_{1} ^{\prime}(0),\;\varphi_{1} (0) = \delta \,\varphi_{1} ^{\prime}(0)} \\ {f_{1} ^{\prime} \to 0,\;g_{1} \to 0,\;\theta_{1} \to 0,\;\varphi_{1} \to 0\quad as\quad \eta \to \eta_{\infty } } \\ \end{array} } \right\}.$$

The analytical solution for the zero – order equations as given in Eqs. (45)–(48) which correspond to the boundary conditions (49) has been analytically constructed as follows:

55 $$f_{0} (\eta ) = \frac{{c_{1} }}{M}e^{M\eta } - \frac{{c_{2} }}{M}e^{ - M\eta } + c_{3} ,$$

56 $$g_{0} (\eta ) = c_{4} e^{M\eta } + c_{5} e^{ - M\eta } ,$$

57 $$\theta_{0} (\eta ) = c_{6} \eta + c_{7} ,$$

and

58 $$\varphi_{0} (\eta ) = c_{8} \eta + c_{9} .$$

To follow the paper easily, the constant $c_{1} ,\;c_{2} , \ldots ,\;c_{9}$ will be moved to the Appendix.

The following are the analytical solutions of the first-order Eqs. (50)–(53) with the required boundary conditions (54):

59 $$f_{1} (\eta ) = \frac{{c_{10} }}{{6M^{3} }}e^{2M\eta } - \frac{{c_{11} }}{{6M^{3} }}e^{ - 2M\eta } + \left[ {\frac{{c_{15} }}{M} + \frac{{c_{12} }}{2M}\left( {\frac{\eta }{M} - \frac{1}{{M^{2} }}} \right)} \right]e^{M\eta } - \left[ {\frac{{c_{16} }}{M} - \frac{{c_{13} }}{2M}\left( {\frac{\eta }{M} + \frac{1}{{M^{2} }}} \right)} \right]e^{ - M\eta } - \frac{{c_{14} }}{{M^{2} }}\eta + c_{19} ,$$

60 $$g_{1} (\eta ) = \frac{{c_{21} }}{{3M^{2} }}e^{2M\eta } + \frac{{c_{22} }}{{3M^{2} }}e^{ - 2M\eta } + \frac{{c_{23} }}{2M}e^{M\eta } - \frac{{c_{24} }}{2M}e^{ - M\eta } \,\, - \frac{{c_{25} }}{{M^{2} }} + c_{26} e^{M\eta } + c_{27} e^{ - M\eta } ,$$

61 $$\theta_{1} (\eta ) = {\text{Pr}} \left[ \begin{gathered} - 2c_{6} \left(\frac{{c_{1} }}{{M^{3} }}e^{M\eta } - \frac{{c_{2} }}{{M^{3} }}e^{ - M\eta } + \frac{{c_{3} }}{2}\eta^{2} \right) + 4\lambda_{1} c_{6} \left(\frac{{c_{1}^{2} }}{{4M^{3} }}e^{2M\eta } - \frac{{c_{2}^{2} }}{{4M^{3} }}e^{ - 2M\eta }\right. \hfill \\ \quad \left. + \frac{{c_{1} c_{3} }}{{M^{2} }}e^{M\eta } + \frac{{c_{2} c_{3} }}{{M^{2} }}e^{ - M\eta } \right) - \frac{1}{2}(N_{B} c_{6} c_{8} + N_{T} c_{6}^{2} )\eta^{2} \hfill \\ \end{gathered} \right] + c_{30} \eta + c_{31} ,$$

62 $$\varphi_{1} (\eta ) = S_{c} c_{8} \left[ \begin{gathered} - 2 \left(\frac{{c_{1} }}{{M^{3} }}e^{M\eta } - \frac{{c_{2} }}{{M^{3} }}e^{ - M\eta } + \frac{{c_{3} }}{2}\eta^{2} \right) + 4\lambda_{2} \left(\frac{{c_{1}^{2} }}{{4M^{3} }}e^{2M\eta } \right. \hfill \\ \quad \left. - \frac{{c_{2}^{2} }}{{4M^{3} }}e^{ - 2M\eta } + \frac{{c_{1} c_{3} }}{{M^{2} }}e^{M\eta } \, + \frac{{c_{2} c_{3} }}{{M^{2} }}e^{ - M\eta } \right) \hfill \\ \end{gathered} \right] + c_{35} \eta + c_{36} .$$

The constants $c_{10} ,\;c_{11} , \ldots ,\;c_{36}$ will be moved to the Appendix to follow the manuscript easily.

Lastly, in view of the HPM, the estimated solution of the non-dimensional velocity components, temperature and nanoparticles concentration may be written as follows:

63 $$\chi (\eta ) = \mathop {\lim }\limits_{q \to 1} \left( {\chi_{0} (\eta ) + q\chi_{1} (\eta ) + \cdots } \right),$$

Consequently, the profiles of these functions can be represented as:

64 $$f^{\prime}(\eta ) = f_{0} ^{\prime}(\eta ) + f_{1} ^{\prime}(\eta ) = \frac{{c_{10} }}{{3M^{2} }}e^{2M\eta } + \frac{{c_{11} }}{{3M^{2} }}e^{ - 2M\eta } + \left( {c_{1} + c_{15} + \frac{{c_{12} }}{2M}} \right)e^{M\eta } + \left( {c_{2} + c_{16} - \frac{{c_{13} }}{2M}} \right)e^{ - M\eta } - \frac{{c_{14} }}{{M^{2} }},$$

65 $$g(\eta ) = g_{0} (\eta ) + g_{1} (\eta ) = \frac{{c_{21} }}{{3M^{2} }}e^{2M\eta } + \frac{{c_{22} }}{{3M^{2} }}e^{ - 2M\eta } + \left( {c_{4} + + c_{26} + \frac{{c_{23} }}{2M}} \right)e^{M\eta } + \left( {c_{5} + c_{27} - \frac{{c_{24} }}{2M}} \right)e^{ - M\eta } \, - \frac{{c_{25} }}{{M^{2} }},$$

66 $$\left. \begin{gathered} \theta (\eta ) = \theta_{0} (\eta ) + \theta_{1} (\eta ) = {\text{Pr}} \left[ - 2c_{6} \left(\frac{{c_{1} }}{{M^{3} }}e^{M\eta } - \frac{{c_{2} }}{{M^{3} }}e^{ - M\eta } + \frac{{c_{3} }}{2}\eta^{2} \right) + \lambda_{1} c_{6} \left(\frac{{c_{1}^{2} }}{{M^{3} }}e^{2M\eta } - \frac{{c_{2}^{2} }}{{M^{3} }}e^{ - 2M\eta }\right.\right. \hfill \\ \quad \left.\left. + \frac{{4c_{1} c_{3} }}{{M^{2} }}e^{M\eta } + \frac{{4c_{2} c_{3} }}{{M^{2} }}e^{ - M\eta } \right) - \frac{1}{2}(N_{B} c_{6} c_{8} + N_{T} c_{6}^{2} )\eta^{2} \right] + (c_{6} + c_{30} )\eta + c_{7} + c_{31} \hfill \\ \end{gathered} \right\},$$

and

67 $$\left. \begin{gathered} \varphi (\eta ) = \varphi_{0} (\eta ) + \varphi_{1} (\eta ) = S_{c} c_{8} \left[ - 2\left(\frac{{c_{1} }}{{M^{3} }}e^{M\eta } - \frac{{c_{2} }}{{M^{3} }}e^{ - M\eta } + \frac{{c_{3} }}{2}\eta^{2} \right) + \lambda_{2} \left(\frac{{c_{1}^{2} }}{{M^{3} }}e^{2M\eta } \right.\right.\hfill \\ \quad \left. \left. - \frac{{c_{2}^{2} }}{{M^{3} }}e^{ - 2M\eta } + \frac{{4c_{1} c_{3} }}{{M^{2} }}e^{M\eta } + \frac{{4c_{2} c_{3} }}{{M^{2} }}e^{ - M\eta } \right)\right] + (c_{8} + c_{35} )\eta + c_{9} + c_{36} \hfill \\ \end{gathered} \right\}.$$

The following section seeks to explore the impacts of the parameter settings in the problem at hand on the various distributions of nanoparticles velocity, heat, and concentrations, with some diagrams to further demonstrate these influences.

## Results and discussion

Under the influence of Stefan blowing, the stationary two-dimensional MHD border layer flow of Reiner–Rivlin nanoparticles over a revolving extending warmed disc is explored. The HPM is utilized to analyze the non-dimensional ODEs (32)–(35) with border restrictions (39).

In order to elucidate the problem physically**,** the results are discussed to demonstrate the effects of numerous restrictions. These factors include the Reiner–Rivlin fluid factor $K$, the Brownian movement limit $N_{B}$, the thermophoresis limitation $N_{T}$, the rate of the stretching constraint $\alpha$, the nanoparticle volume fraction slip parameter *δ*, the Schmidt numeral $S_{c}$ and the Prandtl number ${\text{Pr}}$. The study at hand concentrates on the impacts of these limitations on the velocity, temperature, nanoparticles profiles, as well as skin-friction $C_{f}$, Nusselt $Nu$ and Sherwood $Sh$ quantities. These distributions are plotted in accordance with the data mentioned in Figs. 2, 3, 4, 5, 6, 7, 8, 9, 10, 11, 12, 13, 14, 15, 16, 17, 18, 19, 20, 21, 22, 23, 24, 25, 26, 27, 28, 29, 30, 31, 32, 33.

**Figure 2:**
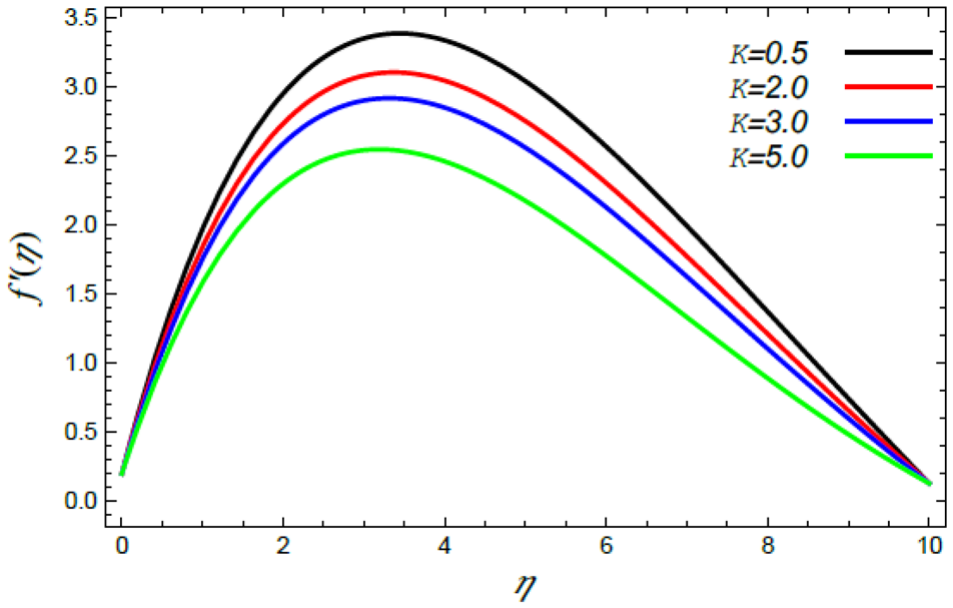
Variation of the radial velocity $f^{\prime}(\eta )$ versus $\eta$ as given in Eq. (64) to illustrate the impact of the material factor $K$.

**Figure 3:**
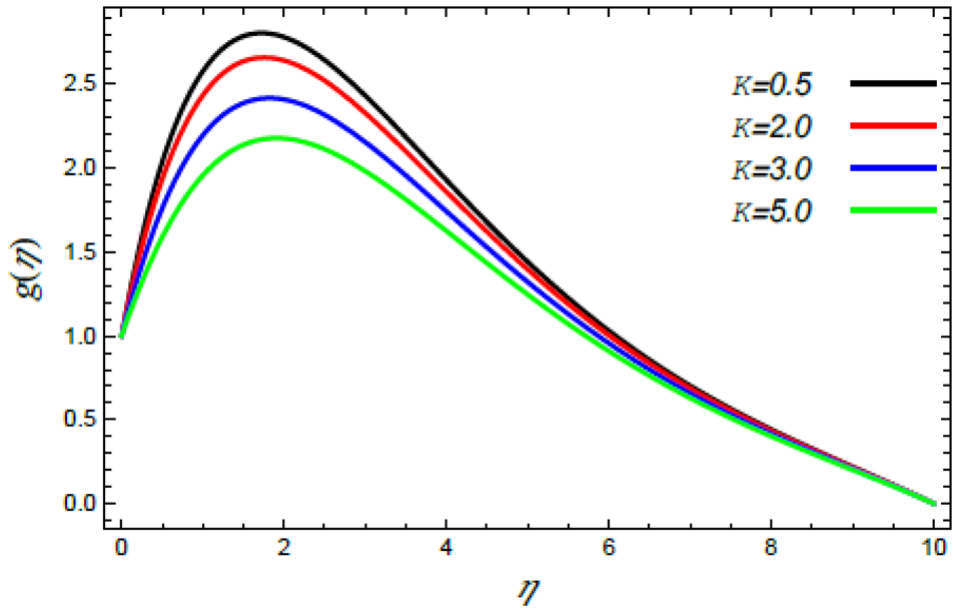
Deviation of the azimuthal speed $g(\eta )$ versus $\eta$ as given in Eq. (65) to illustrate the impact of the material factor $K$.

**Figure 4:**
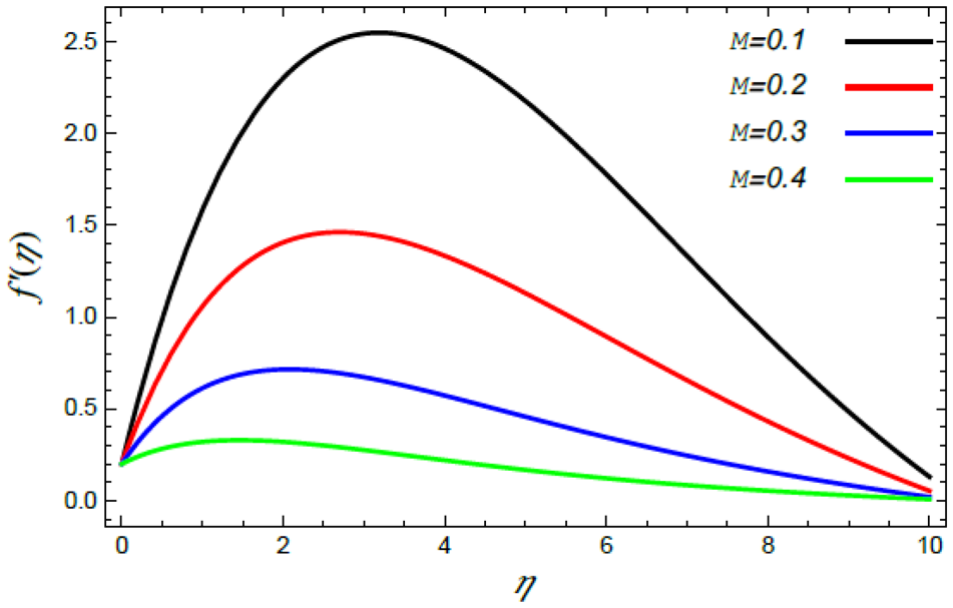
Deviation of the radial velocity $f^{\prime}(\eta )$ versus $\eta$ as given in Eq. (64) to illustrate the impact of the magnetic factor $M$.

**Figure 5:**
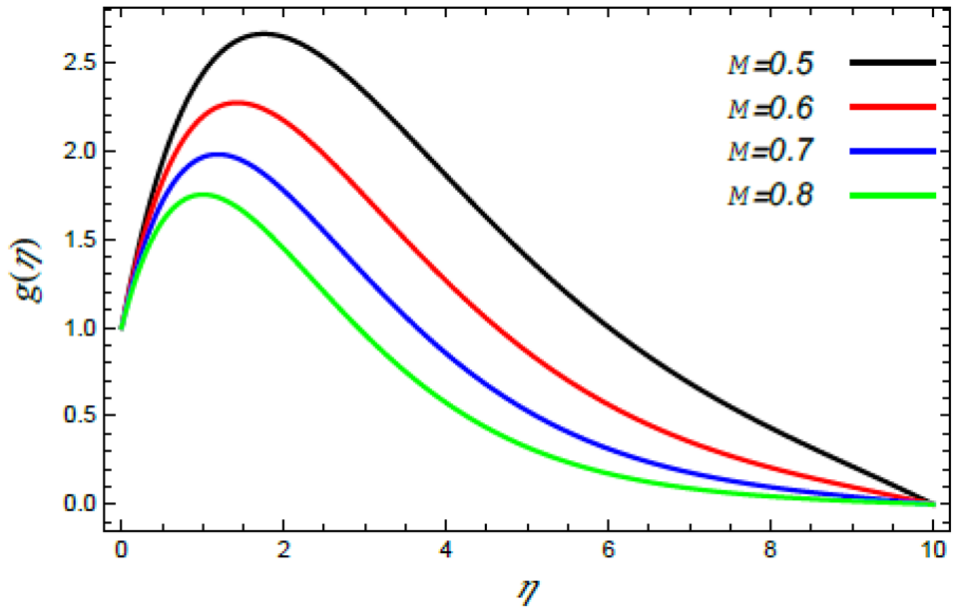
Deviation of the azimuthal speed $g(\eta )$ versus $\eta$ as given in Eq. (65) to illustrate the impact of the magnetic factor $M$.

**Figure 6:**
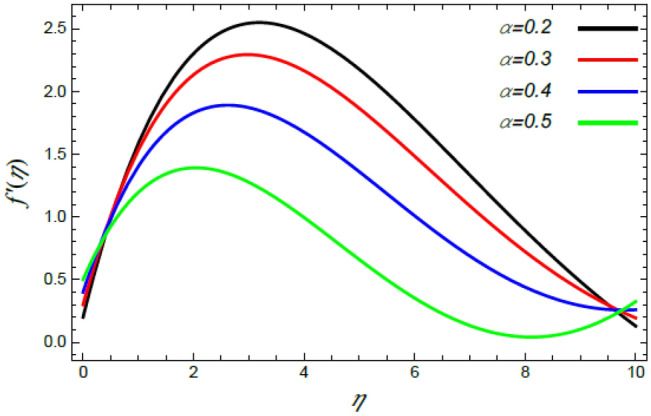
Deviation of the radial speed $f^{\prime}(\eta )$ versus $\eta$ as given in Eq. (64) to illustrate the influence of the stretching factor $\alpha$.

**Figure 7:**
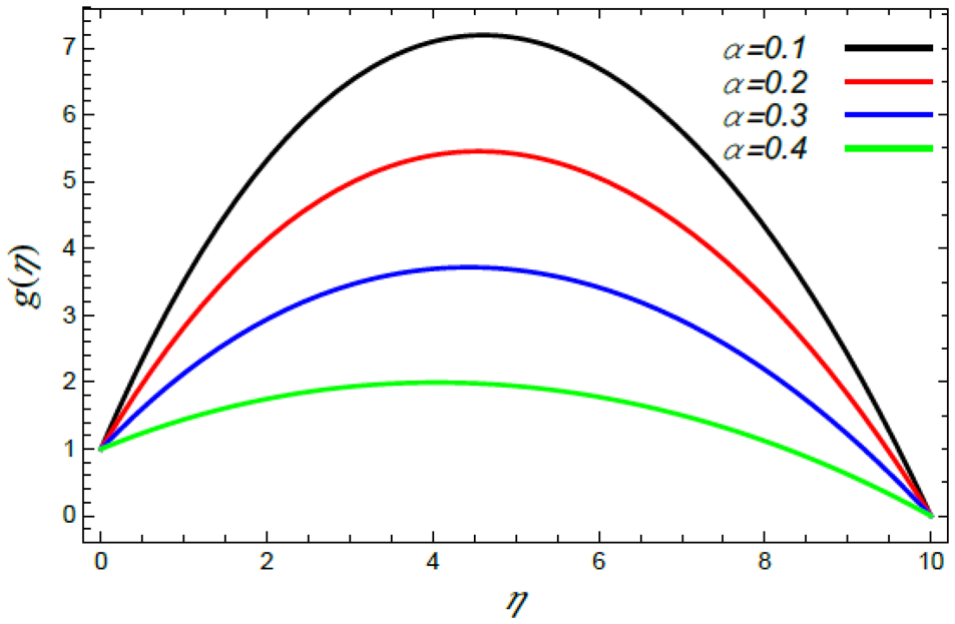
Deviation of the azimuthal speed $g(\eta )$ versus $\eta$ as given in Eq. (65) to illustrate the impact of the stretching factor $\alpha$.

**Figure 8:**
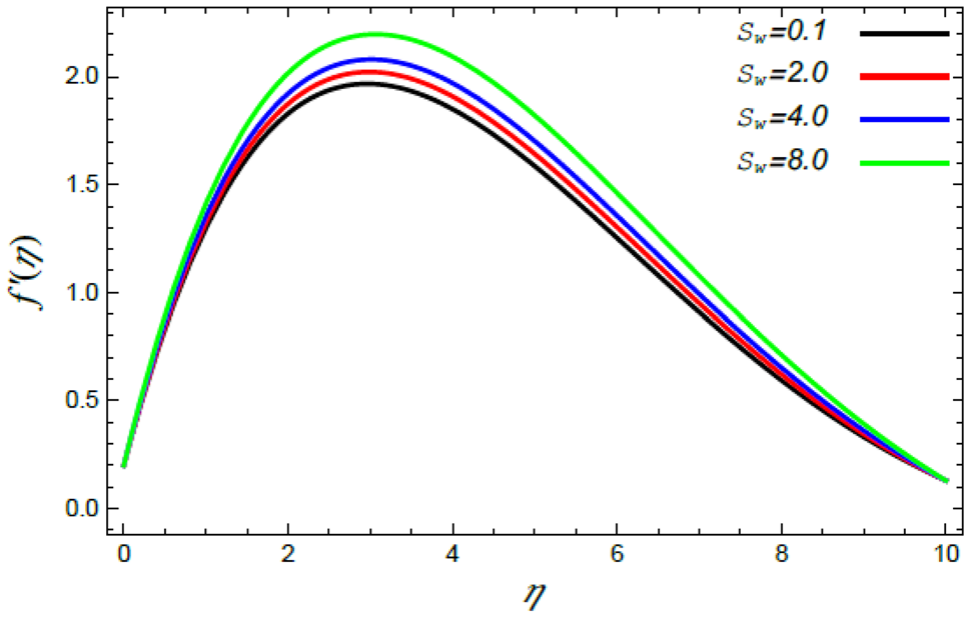
Deviation of the radial speed $f^{\prime}(\eta )$ versus $\eta$ as given in Eq. (64) to illustrate the impact of the Stefan blowing factor $S_{w}$.

**Figure 9:**
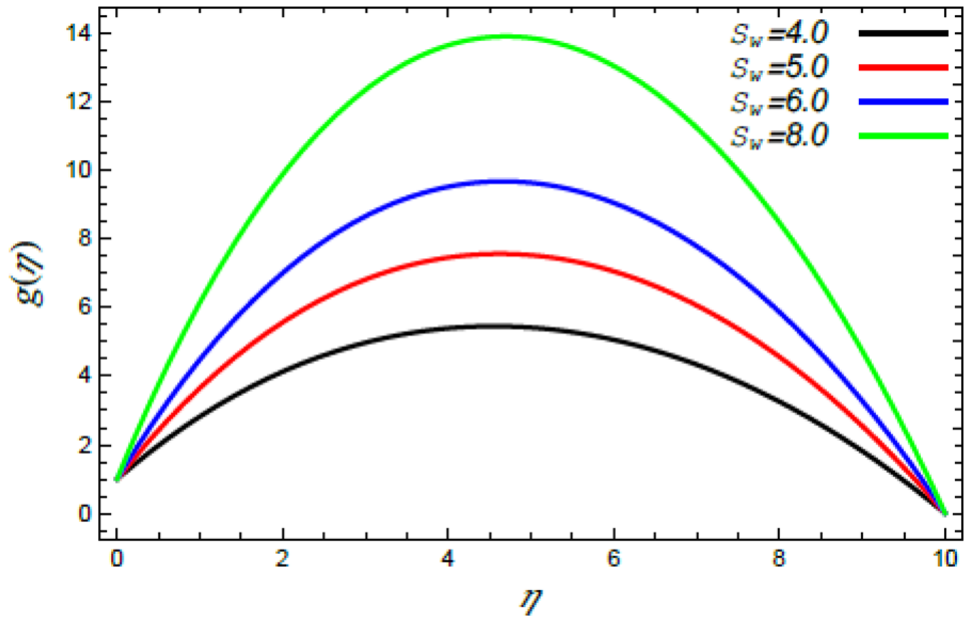
Deviation of the azimuthal velocity $g(\eta )$ versus $\eta$ as given in Eq. (65) to illustrate the impact of the Stefan blowing factor $S_{w}$.

**Figure 10:**
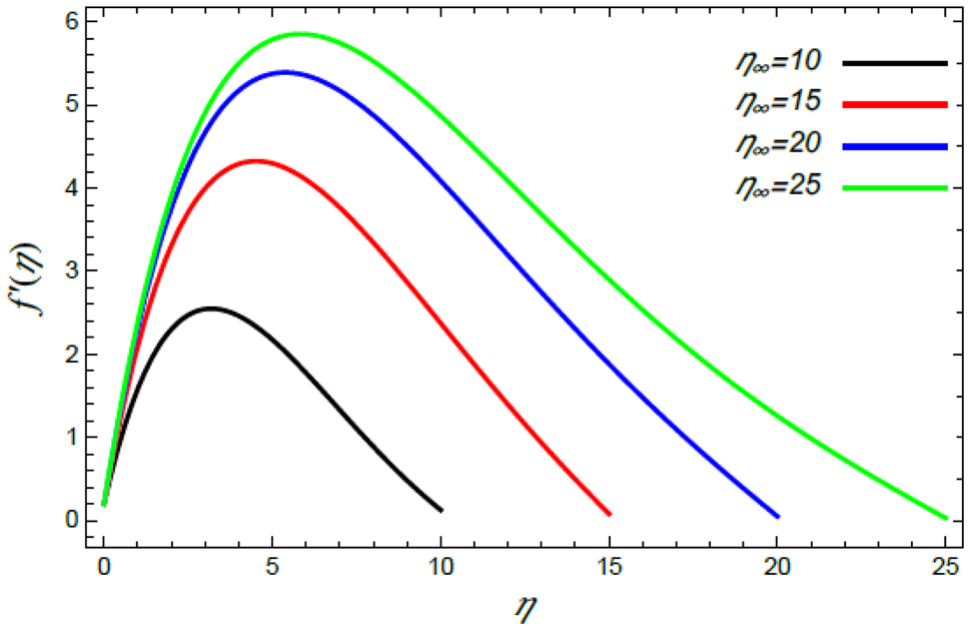
Variation of the radial velocity $f^{\prime}(\eta )$ versus $\eta$ as given in Eq. (64) to depict the effect of $\eta_{\infty }$.

**Figure 11:**
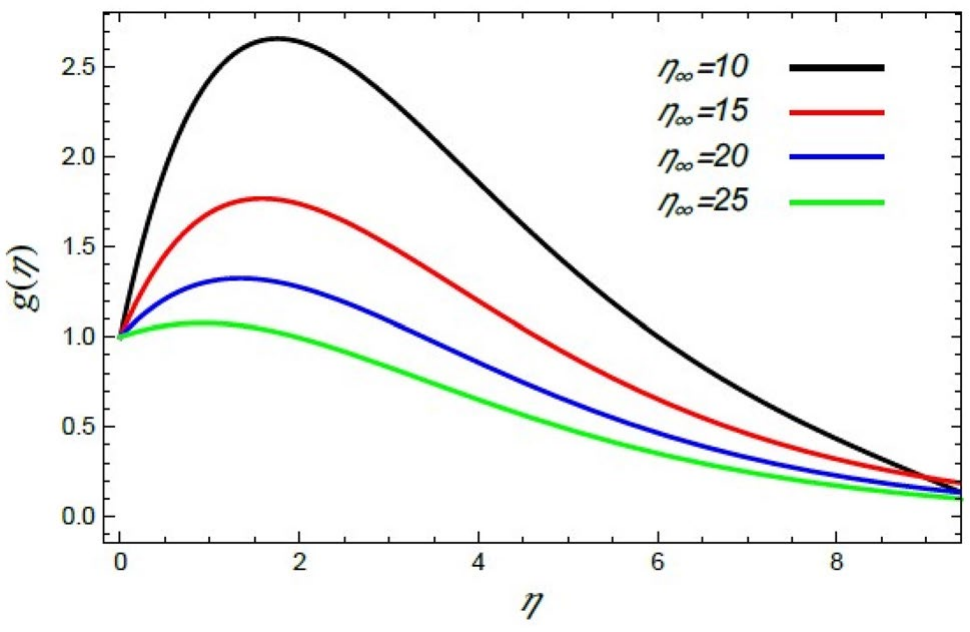
Variation of the azimuthal velocity $g(\eta )$ versus $\eta$ as given in Eq. (65) to depict the effect of $\eta_{\infty }$.

**Figure 12:**
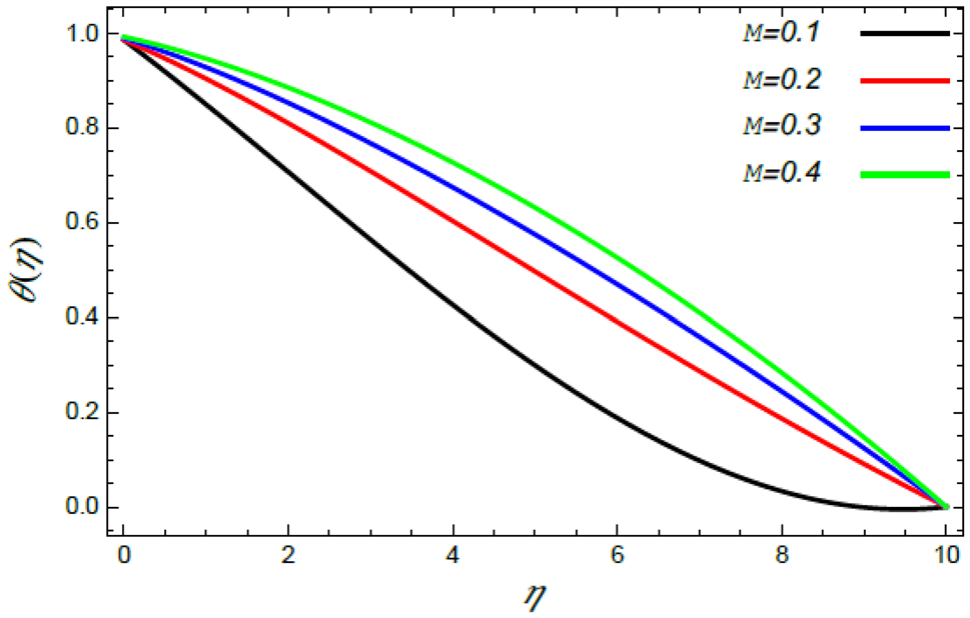
Deviation of the temperature profile $\theta (\eta )$ versus $\eta$ as given in Eq. (66) to illustrate the impact of the magnetic factor $M$.

**Figure 13:**
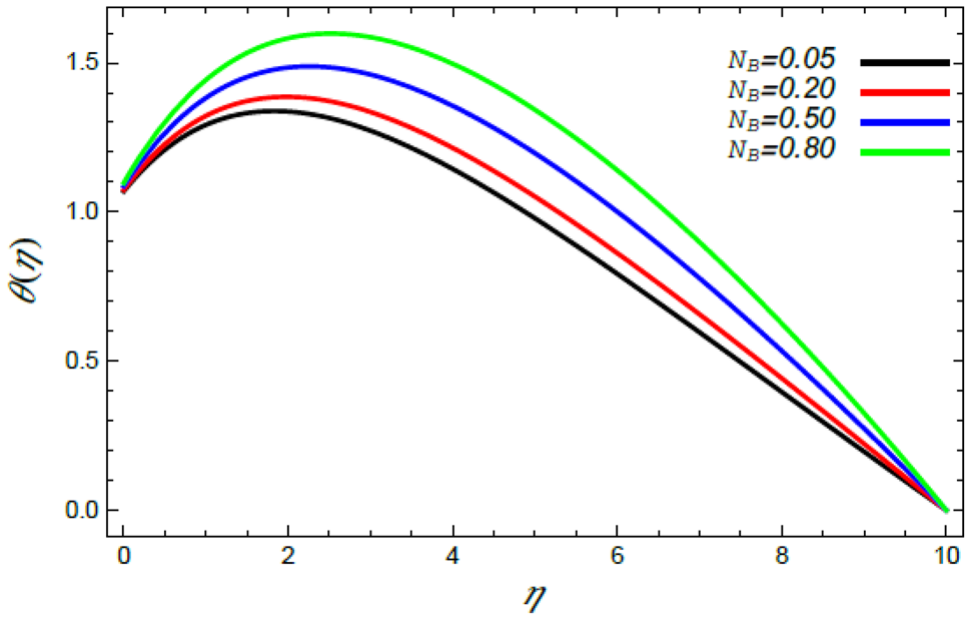
Deviation of the heat profile $\theta (\eta )$ versus $\eta$ as given in Eq. (66) to illustrate the impact of the Brownian motion factor $N_{B}$.

**Figure 14:**
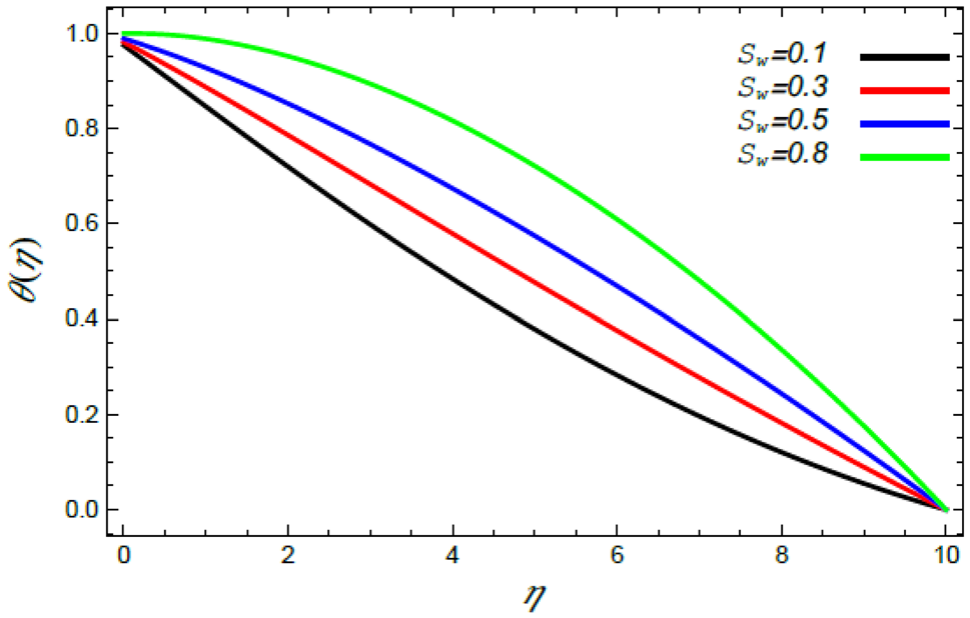
Deviation of the heat profile $\theta (\eta )$ versus $\eta$ as given in Eq. (66) to illustrate the impact of the Stefan blowing factor $S_{w}$.

**Figure 15:**
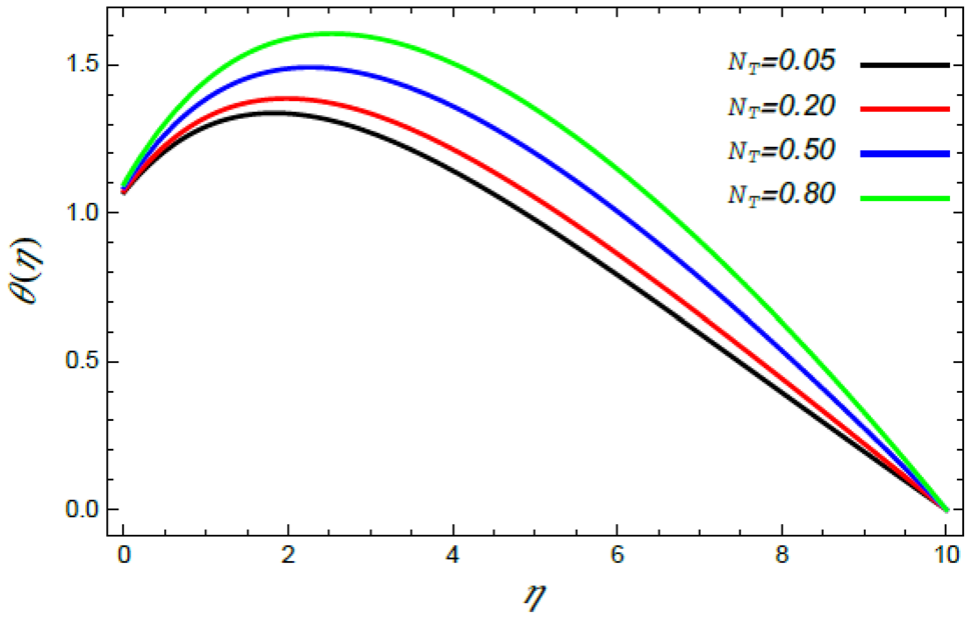
Deviation of the heat profile $\theta (\eta )$ versus $\eta$ as given in Eq. (66) to illustrate the impact of the thermophoresis factor $N_{T}$.

**Figure 16:**
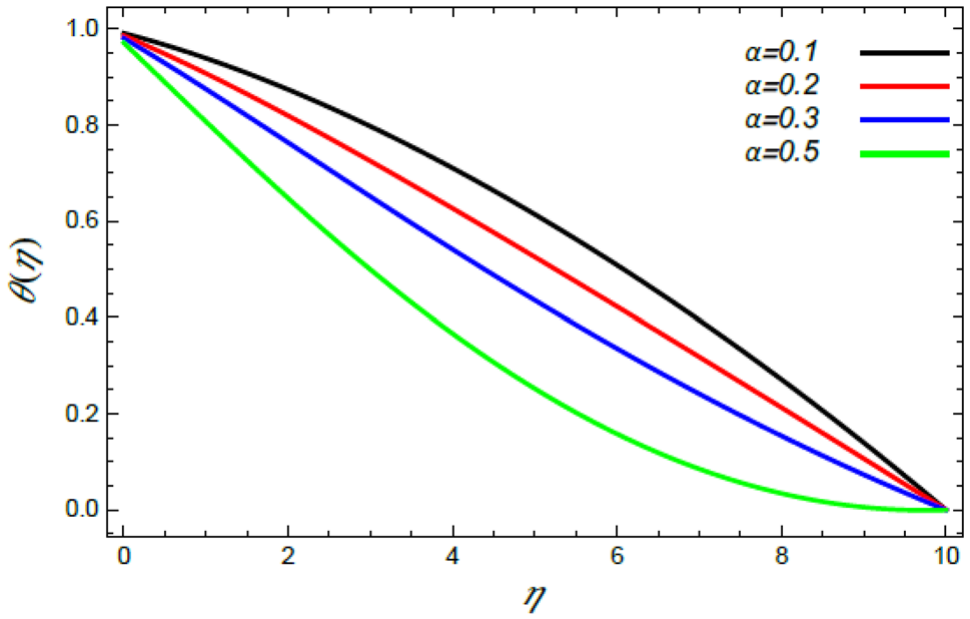
Deviation of the heat profile $\theta (\eta )$ versus $\eta$ as given in Eq. (66) to illustrate the impact of the stretching factor $\alpha$.

**Figure 17:**
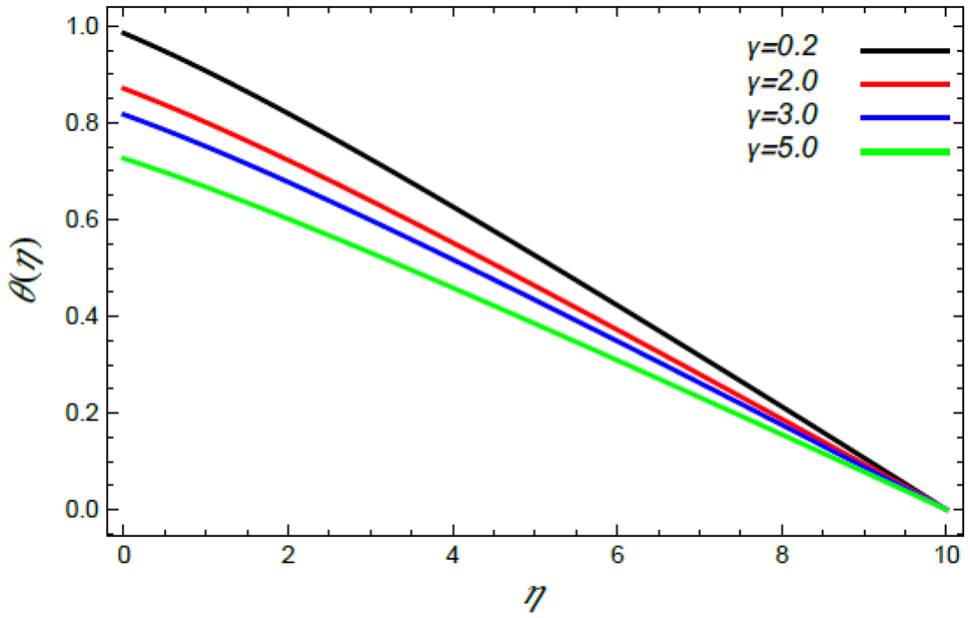
Deviation of the heat profile $\theta (\eta )$ versus $\eta$ as given in Eq. (66) to illustrate the impact of the thermal slip factor $\gamma$.

**Figure 18:**
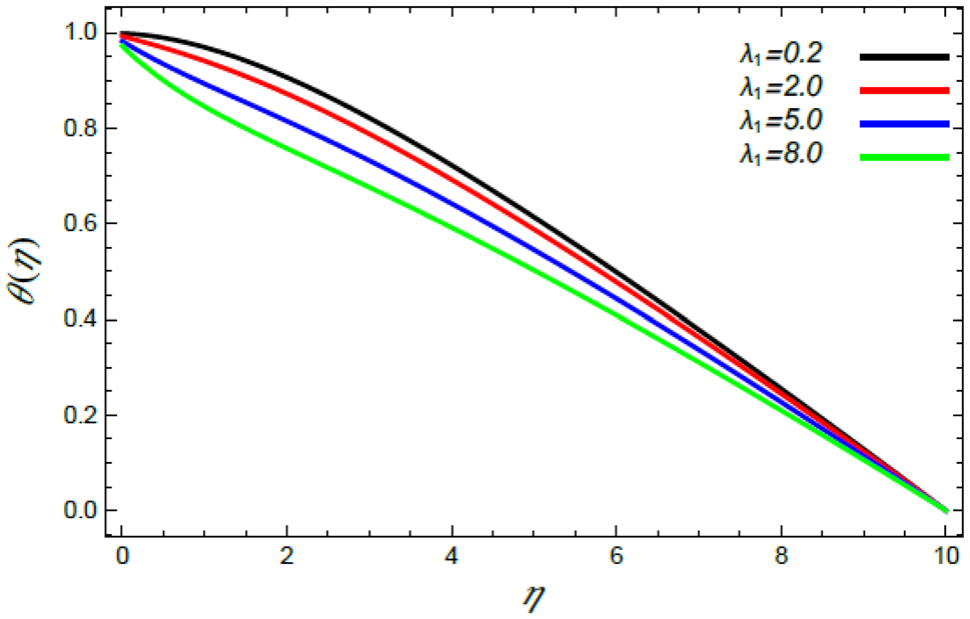
Deviation of the heat profile $\theta (\eta )$ versus $\eta$ as given in Eq. (66) to illustrate the impact of the heat dispersion factor $\lambda_{1}$.

**Figure 19:**
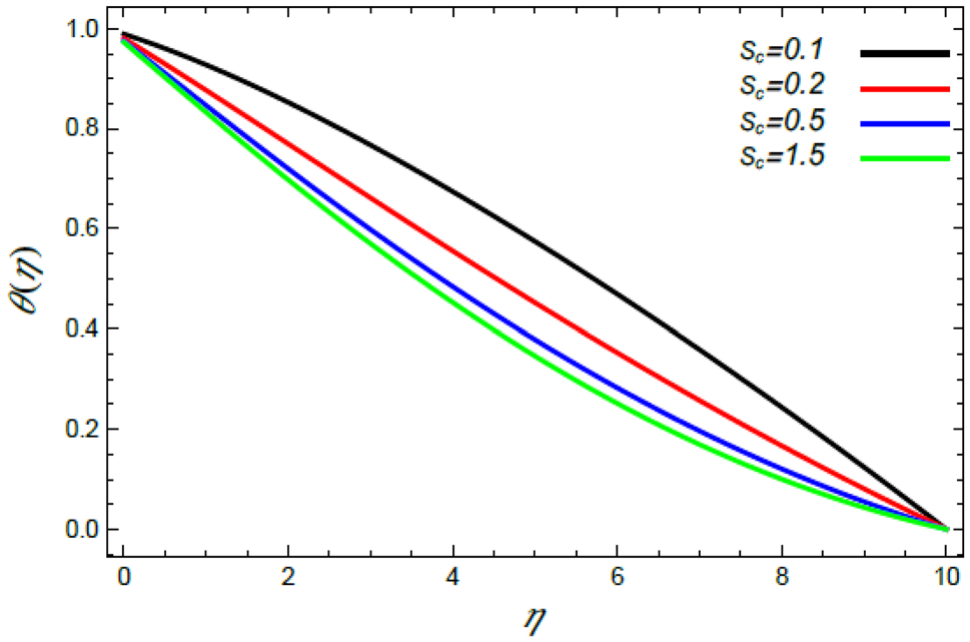
Deviation of the heat profile $\theta (\eta )$ versus $\eta$ as given in Eq. (66) to illustrate the impact of the Schmidt numeral $S_{c}$.

**Figure 20:**
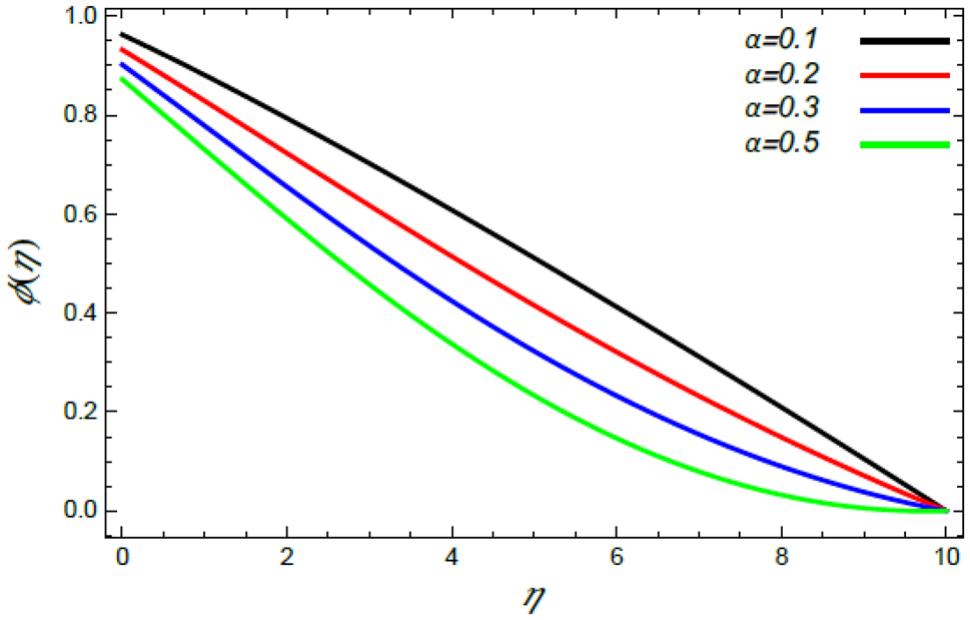
Deviation of the nanoparticle profile $\varphi (\eta )$ versus $\eta$ as given in Eq. (67) to illustrate the impact of the stretching factor $\alpha$.

**Figure 21:**
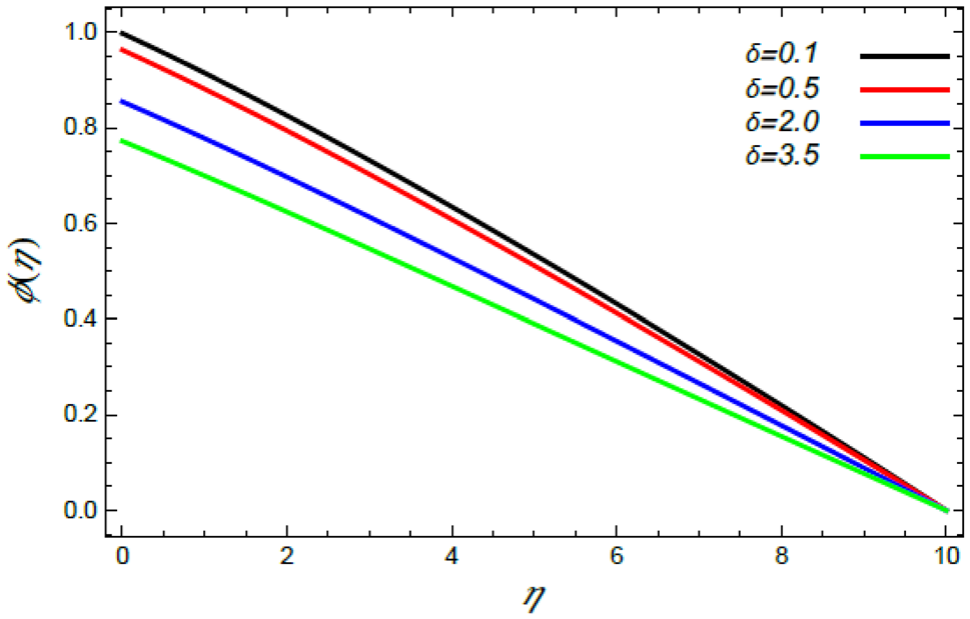
Deviation of the nanoparticle profile $\varphi (\eta )$ versus $\eta$ as given in Eq. (67) to illustrate the impact of the slip factor $\delta$.

**Figure 22:**
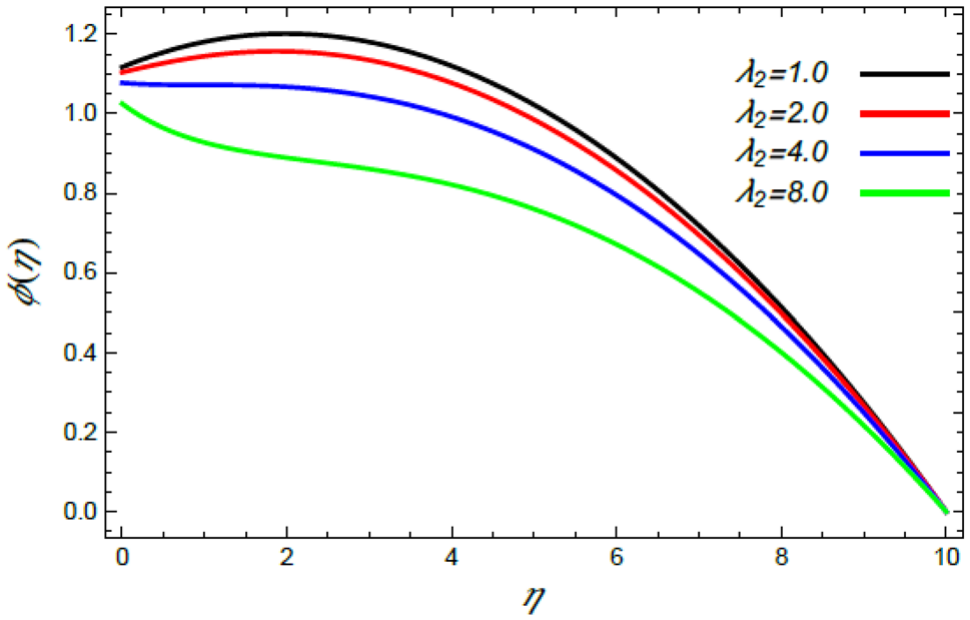
Deviation of the nanoparticle profile $\varphi (\eta )$ versus $\eta$ as given in Eq. (67) to illustrate the impact of the Deborah factor $\lambda_{2}$.

**Figure 23:**
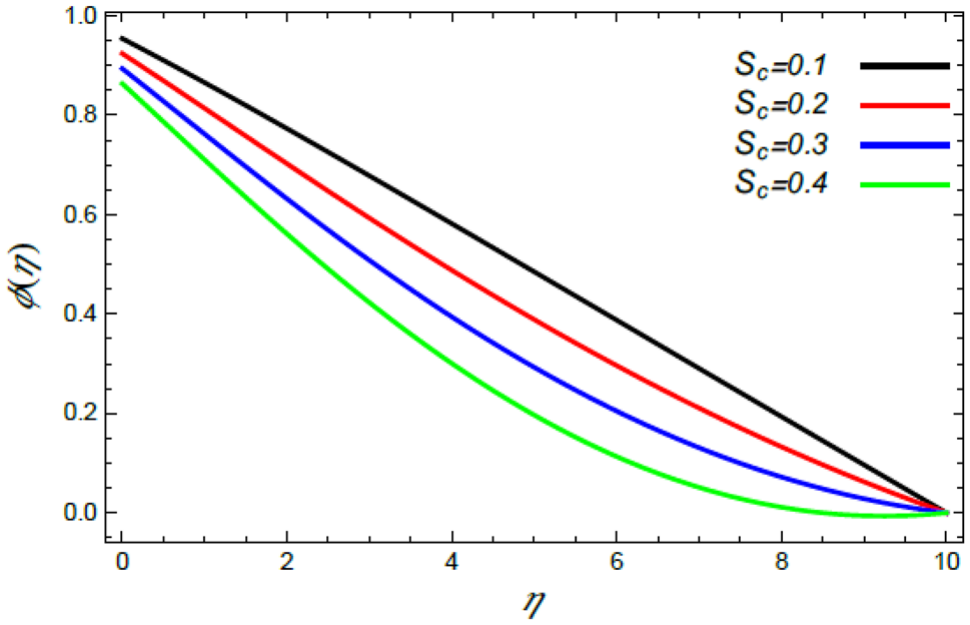
Variation of the nanoparticle concentration $\varphi (\eta )$ versus $\eta$ as given in Eq. (67) to illustrate the impact of the Schmidt numeral $S_{c}$.

**Figure 24:**
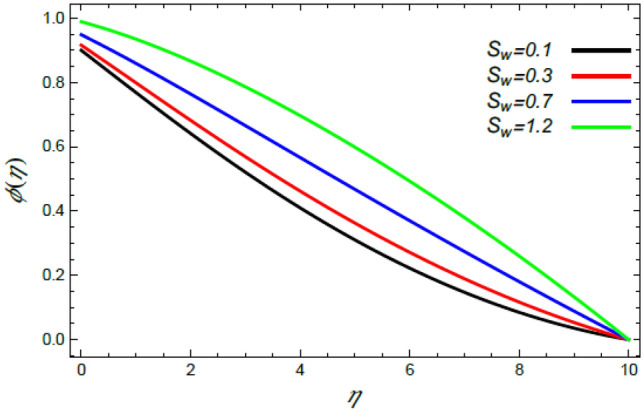
Deviation of the nanoparticle profile $\varphi (\eta )$ versus $\eta$ as given in Eq. (67) to illustrate the impact of the Stefan blowing factor $S_{w}$.

**Figure 25:**
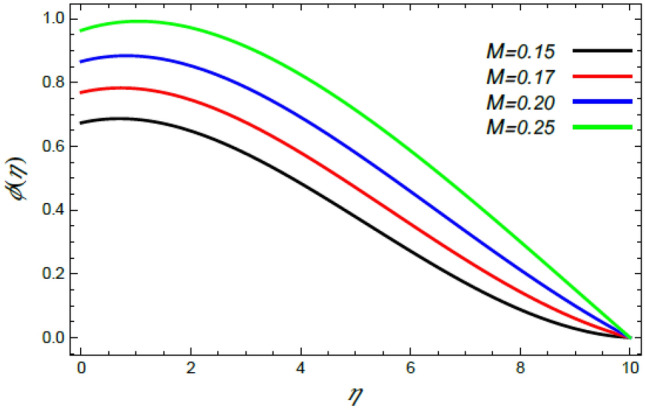
Deviation of the nanoparticle profile $\varphi (\eta )$ versus $\eta$ as given in Eq. (67) to illustrate the impact of the magnetic parameter $M$.

**Figure 26:**
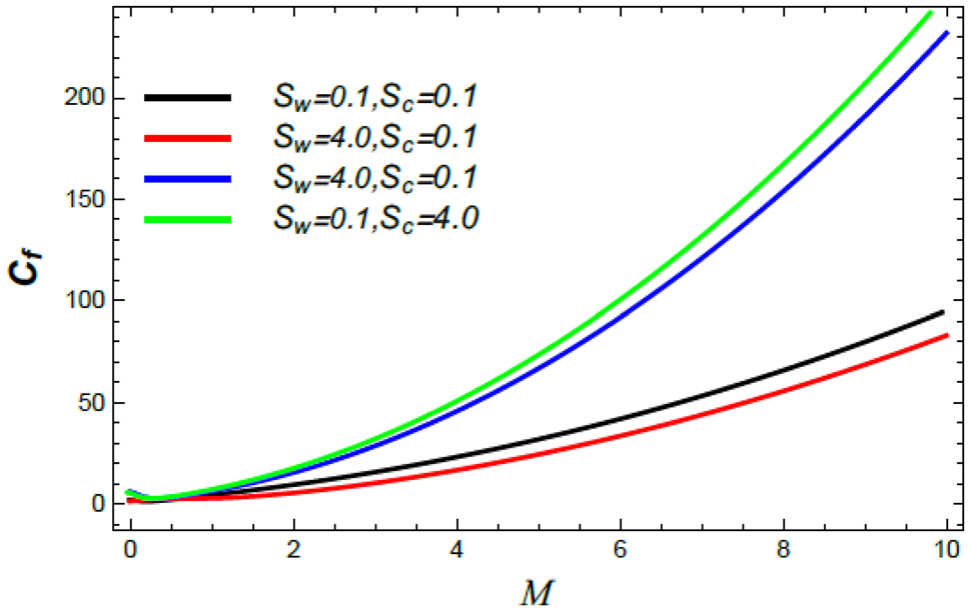
Skin friction coefficient versus $M$ as given in Eq. (36) to depict the effect of the Stefan blowing parameter $S_{w}$ and Schmidt number $S_{c}$.

**Figure 27:**
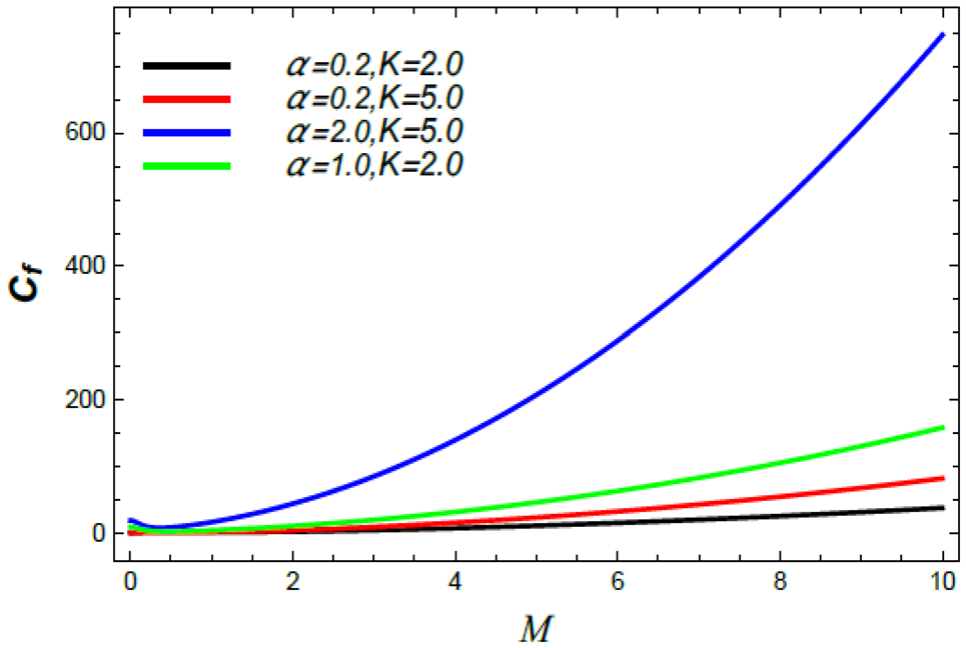
Skin friction coefficient versus $M$ as given in Eq. (36) to illustrate the impact of the stretching factor $\alpha$ and the material factor $K$.

**Figure 28:**
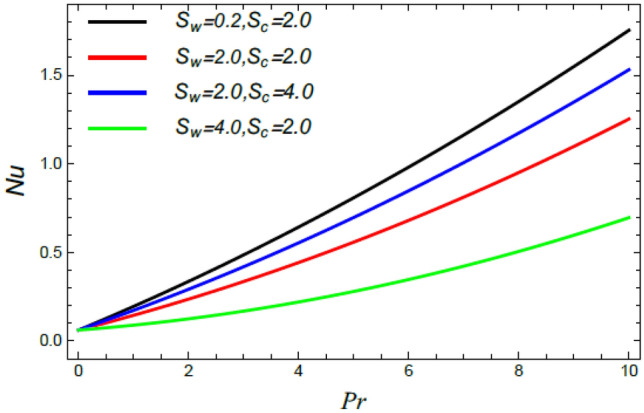
Nusselt number versus ${\text{Pr}}$ as given in Eq. (37) to depict the effect of the Stefan blowing factor $S_{w}$ and Schmidt numeral $S_{c}$.

**Figure 29:**
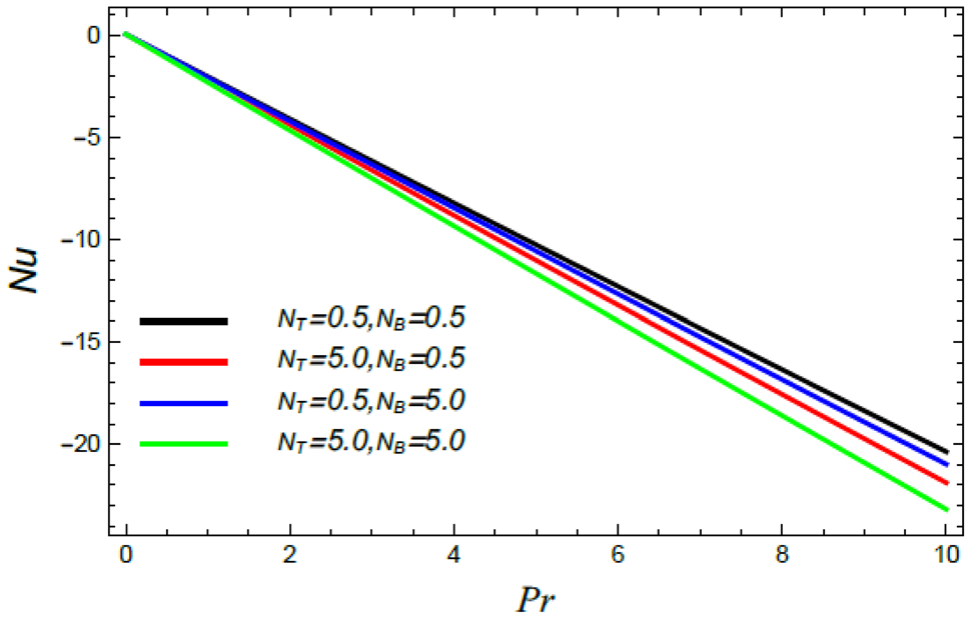
Nusselt number versus ${\text{Pr}}$ as given in Eq. (37) to depict the impact of the Brownian motion factor $N_{B}$ and the thermophoresis factor $N_{T}$.

**Figure 30:**
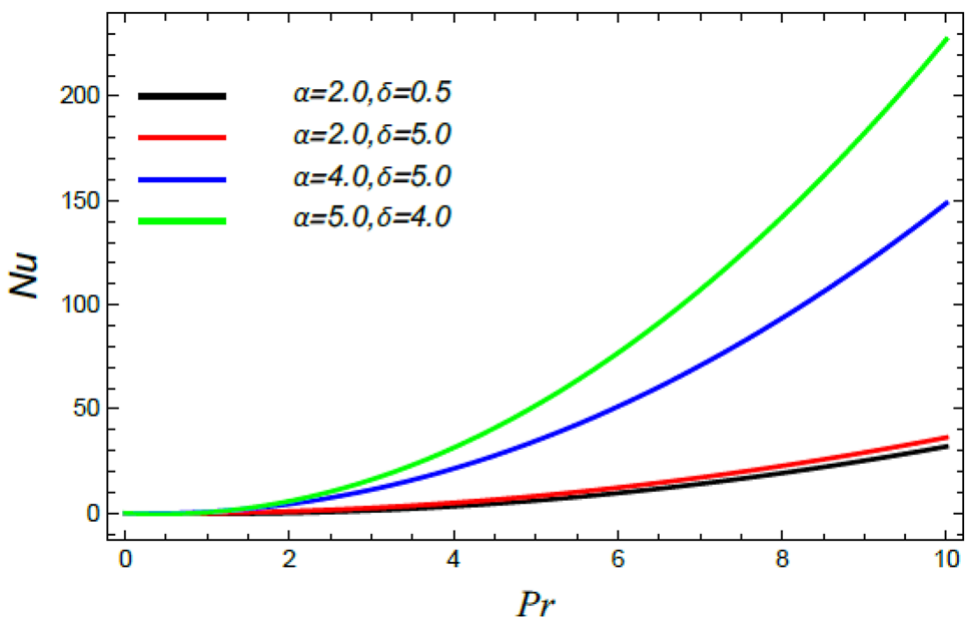
Nusselt numeral versus ${\text{Pr}}$ as given in Eq. (37) to illustrate the impact of the stretching factor $\alpha$ and the slip parameter $\delta$.

**Figure 31:**
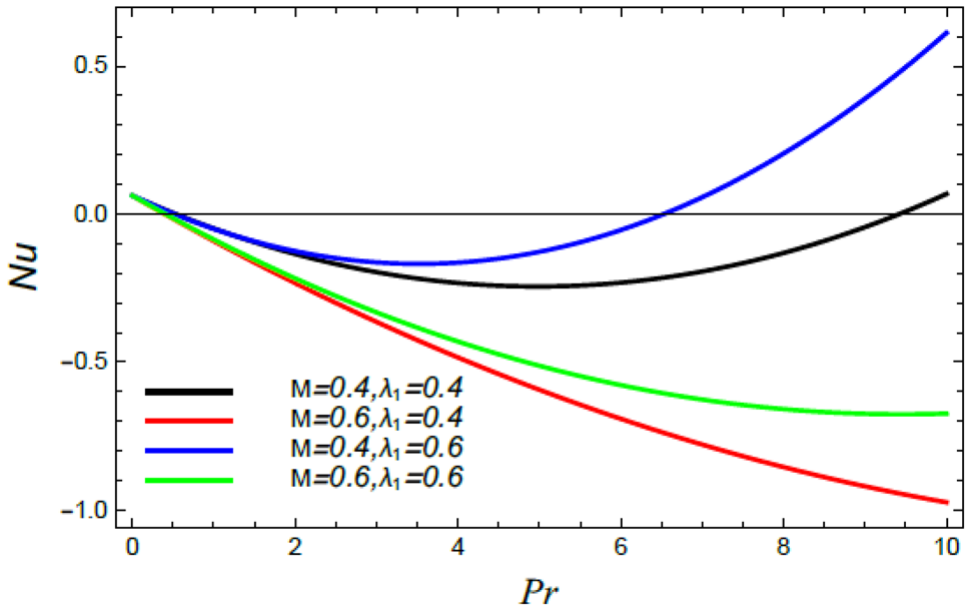
Nusselt number versus ${\text{Pr}}$ as given in Eq. (37) to depict the impact of the magnetic factor $M$ and the heat dispersion parameter $\lambda_{1}$.

**Figure 32:**
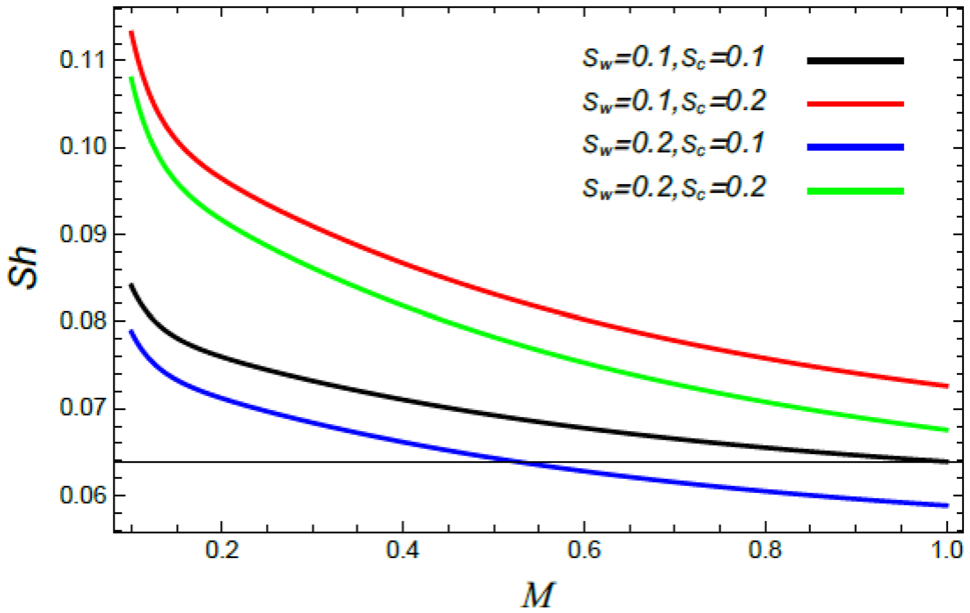
Sherwood number versus $M$ as given in Eq. (38) to illustrate the impact of the Stefan blowing factor $S_{w}$ and Schmidt number $S_{c}$.

**Figure 33:**
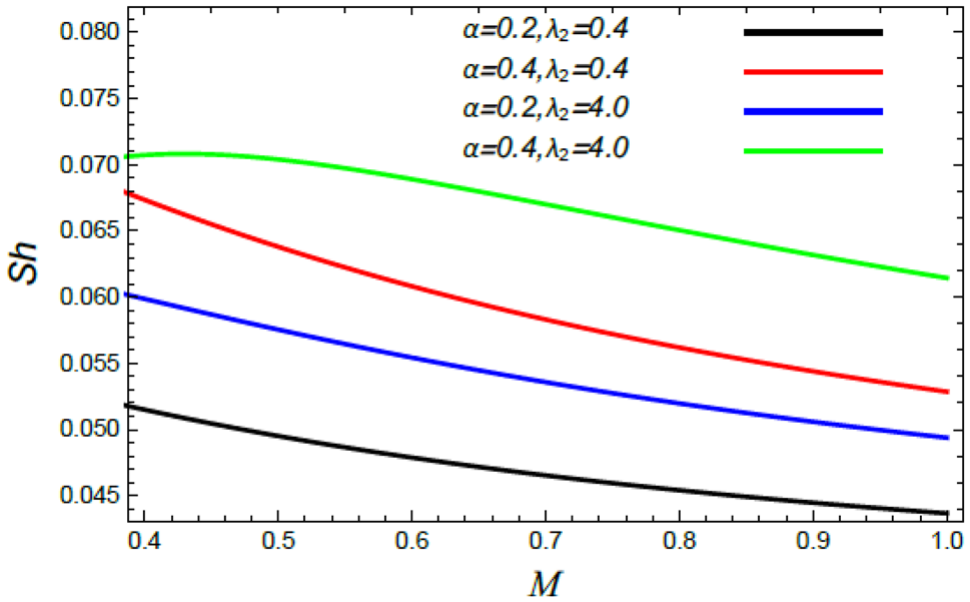
Sherwood number versus $M$ as given in Eq. (38) to illustrate the impact of the stretching factor $\alpha$ and Deborah parameter $\lambda_{2}$.

For more practicality, the above-mentioned distributions are graphed along with the non-dimensional parameter $\eta$ for some standard values of the above factors, which change according to the factor illustrated in every diagram, as follows:

$${\text{Pr}} = 0.7,\,\,S_{w} = 4,\,\delta = 0.5\,,\,\gamma = 0.2,\,M = 0.5,\,N_{B} = 0.3,\,N_{T} = 0.5,\,\,\alpha = 0.2,\,K = 5,\,\lambda_{1} = 0.2,\lambda_{2} = 0.4\,,\,\eta_{\infty } = 10\,\,{\text{and}}\,\,\,Sc = 0.5.$$

### Speed profile

The speed profile is of a considerable interest when examining fluid motion, where it gives many details about the flow field. Therefore, Figs. 2, 3, 4, 5 are plotted to display the changes of the radial as well as azimuthal velocities versus the axis of the disk for various standards of the substantial limitation of the Reiner–Rivlin fluid $K$ and the magnetic factor $M$. It is found that the radial and azimuthal velocities increase near the disk and attain maximum values at a short distance away, and then they decrease as they move far away from it to vanish at the free stream. In details, it is understood from Figs. 2 and 3 that the radial and azimuthal velocities decrease with the rise in the value of $K$. Physically, the non-Newtonian factor $K$, as an amount of an additional viscosity of the liquid, increases the viscosity, and hence decreases the fluid velocity. Figures 4 and 5 indicate that the radial and azimuthal velocities drop by the rise of the magnetic factor $M$. Materially, this happens owing to the development of the Lorentz force that obstructs the liquid movement; therefore, the fluid velocity decreases. These results are in accord with those earlier obtained^2,3,35,41^.

Figures 6 and 7 display the profiles of the radial and azimuthal velocities for diverse standards of the rate of the stretching factor $\alpha$. Figure 6 shows that there is a dual nature in the radial velocity $f^{\prime}(\eta )$ with the rise of $\alpha$, where $f^{\prime}(\eta )$ is enhanced at small values of $\eta$; whereas it decreases at large values of $\eta$. Naturally, nearest the disc, the rate of the stretching parameter improves the radial velocity due to the dependence of stretching of the surface on the radius $r$. After that, this effect is reversed when moving away from the disc. This result corresponds to that previously described^35,42,43^. By contrast, since the azimuthal velocity takes the tangential direction, this dual effect does not occur. Therefore, the growth in the rate of the extending parameter yields a decrease in the azimuthal speed as seen from Fig. 7. This is similar with the preceding depicted findings^35,43^.

Figures 8 and 9 demonstrate the influence of the Stefan blowing limitation $S_{w}$ on the radial and tangential velocities. It is concluded that as the Stefan blowing limitation $S_{w}$ grows, both the radial velocity as well as the tangential velocity increase. Physically, this is due to the fact that the blowing effect causes more strong movement of the fluid, and eventually a great amount of the fluid moves faster in the radial and tangential directions sufficiently away from the disc. This finding is consistent with that previously illustrated^35,42,44^. Lattif et al.^42^ contended that the mutual impact of the rising blowing factor and the stretching stricture cause the radial speed distribution to rise. As a conclusion, the Stefan blowing effect outweighs that of the rate of the stretching parameter.

Numerous works related to the border sheet of non-Newtonian liquid flows, as given in Refs.^2,35,41,42,45^, numerically investigated all the physical distributions. These works couldn’t take infinity greater than 10 in the best cases. Fortunately, in the analytical solutions of the present study, we could take it as larger values $\eta_{\infty }$, where $\eta_{\infty }$ is an arbitrary number. Consequently, the current study scrutinizes the effect of $\eta_{\infty }$ on all the previous physical distributions. Furthermore, the improvement in $\eta_{\infty }$ supports the accidental movement of the nanoparticles, and henceforth it improves the radial motion and reduces the tangential motion. Accordingly, the growth of $f^{\prime}(\eta )$ and drop of $g(\eta )$ with the augmentation of $\eta_{\infty }$ are illustrated in Figs. 10 and 11, respectively. These results correspond to those recently obtained by Sabu et al.^2^.

### Temperature distribution

As shown in the methodology of the problem, the current work investigates the MHD Reiner–Rivlin nanofluid. Accordingly, this mechanism has enhanced the heat transfer rate. Therefore, Figs. 12 and 13 are designed to confirm this imperative phenomenon, where heat distribution rises by the rise of both the magnetic factor $M$ and the Brownian movement parameter $N_{B}$. Just as before, the magnetic strength produces a Lorentz force that contradicts movement, which in turn improves the thermal diffusion as found in Fig. 12. This conclusion is consistent with Mabood et al.^35^ and Alebraheem and Ramzan^41^. In addition, the Brownian movement factor $N_{B}$, as a measure of the random motion of the nanoparticles, raises the temperature in the neighborhood of the boundary layer as observed by Fig. 13. Physically, the random or accidental motion of particles is a logic reason for the rise of heat of the fluid because of the growth of the friction force between particles. This finding is in accord with the earlier result^46,47^.

Figures 14 and 15 show the enhancement in the temperature due to the growth in the Stefan blowing factor $S_{w}$ and the thermophoresis factor $N_{T}$. Actually, the growth of the blowing parameter causes more collision between nanofluid particles, where the heat of the liquid rises as a result of this collision, which elevates the temperature of the liquid as indicated by Fig. 14. Moreover, the thermophoresis factor $N_{T}$ increases the flow of nanoparticles from the warm surface of the ambient liquid, which yields higher heat in the vicinity of the border sheet as shown in Fig. 15. These findings are consistent with the results of^2,41,42^.

Figures 16 and 17 show the relation between heat and both the stretching factor $\alpha$ and the thermal slip factor $\gamma$, correspondingly. It is revealed that with the rise of $\alpha$ and $\gamma$ a reduced effect on the thermal dispersal is found. Really, the increase of $\alpha$ leads to a wide dispersion of heat since the extending superficial of the ambient fluid develops**,** which brings about a reduction in the liquid heat as depicted in Fig. 16. Figure 17 shows the same performance of $\gamma$, where the thermal slip parameter is a measure of thermal change at the edge. Therefore, it leads to a reduction in the liquid heat from the disc temperature owing to the existence of thermal opposition. These conclusions are parallel to those obtained by Sabu et al.^2^ and Mabood et al.^35^.

Figures 18 and 19 show the reduction of heat transfer in light of the increase of both the Deborah numeral owing to heat dispersion $\lambda_{1}$ and the Schmidt numeral $S_{c}$. Physically, materials that have low relaxation times flow easily, which means that the thermal transfer in the fluid with the increase of the Deborah number $\lambda_{1}$ needs additional time to transmit temperature in the vicinity of liquid elements. Therefore, heat reduces as $\lambda_{1}$ increases, as shown in Fig. 18. This result agrees with that earlier obtained^3,35,43^. Finally, the increase of the Schmidt numeral $S_{c}$, as the ratio between momentum and mass diffusivity, means a reduction in the molecular diffusivity and the random motion of molecules, which yields a decrease in the liquid temperature, as indicated by Fig. 19. The combined effects of those two parameters cause a reduction in the temperature profile as stated by Lattif et al.^42^.

### Nanoparticles volume fraction distribution

In what follows, an interesting phenomenon concerning the nanoparticles volume fraction $\varphi$ is displayed throughout Figs. 20, 21, 22 and 23 to illustrate the effects of the limitations: the rate stretching parameter $\alpha$, the nanoparticles volume fraction slip parameter $\delta$, the Deborah numeral owing to the mass dispersion $\lambda_{2}$ and the Schmidt numeral $S_{c}$, correspondingly. As shown in Fig. 20, the distribution $\varphi$ decreases with the increase of the value of the parameter $\alpha$. Physically, as $\alpha$ rises, an extensive mass diffuses from the stretching sheet into the surrounding liquid. Henceforth, it produces nanoparticles scattering away from the boundary surface**,** and brings about a reduction of the nanoparticles concentration. The growth in the factor $\delta$ provides a objective justification to the decrease in $\varphi$, where the nanoparticles accelerate in their random flow with the increase of $\delta$, as demonstrated in Fig. 21. Figure 22 shows the impact of the factor $\lambda_{2}$ on the $\varphi$ profile. It is found that the profile of $\varphi$ decreases through the rise of $\lambda_{2}$. Actually, the increase of $\lambda_{2}$ means more time to diffuse mass to the vicinity fluid; therefore, the nanoparticles volume fraction $\varphi$ minimizes through the flow. The increase of Schmidt numeral $S_{c}$ reduces the nanoparticles volume fraction, as displayed in Fig. 23, owing to the fact that the Schmidt numeral is the proportion of momentum to mass diffusivity. Consequently, mass diffusivity diminishes for large values of $S_{c}$, which requires a reduction of $\varphi$. All of these findings are consistent with those of^2,35^.

Finally, Figs. 24 and 25 show the impact of both parameters $M$ and $S_{w}$ on the nanoparticles volume fraction $\varphi$. Figure 24 indicates that $\varphi$ rises with the rise of the blowing factor $S_{w}$. This occurs owing to the growing of particles diffusion by the blowing factor, which means an enhancement of $\varphi$. This outcome is in accord with Mabood et al.^35^ and Beg et al.^46^. In addition, Fig. 25 indicates that the $\varphi$ distribution has more increasing rate with the induced magnetic parameter $M$. The speed profile decreases with the intensity magnetic force, and this slow motion leads to more accumulation of nanoparticles and hence an increase in $\varphi$. The same behavior was observed by Mabood et al.^35^.

### Skin friction, Nusselt and Sherwood parameters

Figures 26, 27, 28, 29, 30, 31, 32 and 33 discuss the influences of diverse factors on the skin friction constant $C_{f}$, Nusselt $Nu$ and Sherwood $Sh$ numbers.

Figures 26 and 27 display the variation of $C_{f}$ versus $M$. It is noticed that the skin friction grows through the rise of $M$. Additionally, Fig. 26 illustrates that the skin friction grows with the growth of the Schmidt numeral $S_{c}$ and drops slightly with the Stefan blowing coefficient. Figure 27 indicates that the skin friction grows through the rise of both the stretching factor $\alpha$ and the material factor $K$. These results are physically expected, owing to the appearances of the intersecting velocity gradients of the Reiner–Rivlin nanofluid sheets, which are produced by the enhanced cross-viscosity. These results agree with those obtained earlier^35,42,43^.

Figures 28, 29, 30 and 31 illustrate the behavior of the Nusselt number $Nu$ versus the Prandtl number ${\text{Pr}}$. It is concluded that the influence of ${\text{Pr}}$ fluctuates in accordance with the values of the other parameters. As observed from all these graphs, the Nusselt number drops by the rise of the Stefan blowing coefficient $S_{w}$, the Brownian movement factor $N_{B}$, the thermophoresis factor $N_{T}$ and $M$; whereas it rises with the rise of the Schmidt numeral $S_{c}$, the stretching parameter $\alpha$, the slip parameter *δ* and Deborah number due to energy diffusion $\lambda_{1}$.

Finally, the behavior of the Sherwood number $Sh$ versus the magnetic parameter $M$ is illustrated throughout Figs. 32 and 33. It is found that the Sherwood numeral $Sh$ decreases with the rise of the magnetic factor $M$, the Stefan numeral $S_{w}$ and the Schmidt numeral $S_{c}$; whereas it improves with the growth of the stretching factor $\alpha$ and the Deborah numeral owing to the mass dispersion $\lambda_{2}$. It is worth mentioning that most of these results are consistent with those observed by Mabood et al.^35^ and Lattif et al.^42^.

## Conclusions

The foremost purpose of this work is to scrutinize the straightforward MHD movement of a non-Newtonian laminar hydrodynamic two-dimensional flow that follows the Reiner–Rivlin nanofluid. The cylindrical coordinates are adequate for describing the problem configuration. The border sheet movement lies down on a revolving disc through a stretched parameter in its radial direction. The results of this model may be valuable in many industrial and engineering applications like SDR as mentioned in section “Problem structure”. The physical phenomenon follows the standard approximations of the boundary layer flow. The plate surface does not promote mass concentration and the Stefan blowing effect due to boundary circumstances. The CC heat and mass diffusion concept has also been exploited. The fundamental partial differential equations are transformed into conventional standard ones via correspondence transformations. Several non-dimensional physical numbers are gained. The analysis is performed in light of the HPM to create an estimated distribution of all typical relevant functions concerning velocity, temperature, and concentration, which simplifies the mathematical manipulation. The main findings of the work may be summarized in the following points:

- In accordance with the profiles of the velocity components, it is observed that the rise of the factor $\,S_{w}$ raises the distribution of these components. Simultaneously, the increase of the parameters $M,\,\alpha \,\,\,{\text{and}}\,\,{\text{K}}$ reduces the velocity distributions.
- Heat transmission is examined to specify the impact of the revealed parameters on this distribution. It is found that the increase of the parameters $\alpha ,\,\lambda_{1} ,\,{\text{and}}\,\,{\text{S}}_{{\text{c}}}$ decreases the energy distribution. On the other hand, the increase of the parameters $M,\,N_{B} ,\,S_{w} \,\,\,{\text{and}}\,\,{\text{N}}_{{\text{T}}}$ improves heat transfer. This means that the usage of magnetized nanofluids has an effective role in the applications that need more improvement in heat exchange.
- The nanoparticles volume fraction is investigated regarding the various physical parameters. It is shown that the increase of the parameters $\,S_{w} \,\,\,{\text{and}}\,\,{\text{M}}$ increases this distribution. Meanwhile, the increase of the parameters $\alpha ,\,\delta ,\,\lambda_{2} \,\,\,{\text{and}}\,\,{\text{S}}_{{\text{c}}}$ decreases it.
- The skin friction, Nusselt and Sherwood numerals are illustrated graphically with the variation of the relevant parameters.

## Supplementary Information

Supplementary Information.

## Data Availability

All data generated or analyzed during this study are included in this manuscript.

## English symbols

$$B_{o}$$: Magnetic induction
$C$: Nanoparticles volume fraction
$$C_{\infty }$$: Ambient nanoparticles
$$C_{r}$$: Disc concentration
$C_{f}$: Coefficient of skin friction
$$D_{B}$$: Brownian diffusivity
$$D{}_{T}$$: Thermophoretic diffusion
$e_{ij}$: Rate of strain (deformation)
$f$: Non-dimensional axial velocity
$f^{\prime}$: Non-dimensional radial velocity
$$T_{r}$$: Disc temperature
$g$: Non-dimensional azimuthal velocity
$\underline{J}$: Current density vector
$K$: Reiner–Rivlin material parameter
$u,\;v,\;w$: Fluid velocity components
$$M$$: Magnetic factor
$$N_{T}$$: Thermophoretic parameter
$N_{B}$: Brownian motion factor
$$Nu$$: Nusselt numeral
$$P$$: Pressure
$${\text{Pr}}$$: Prandtl number
$\underline{Q}$: Heat change vector
$${\text{Re}}$$: Reynolds number
$T_{\infty }$: Ambient fluid temperature
$S_{w}$: Stefan blowing parameter
$$S_{c}$$: Schmidt parameter
$$Sh$$: Sherwood number
$$T$$: Fluid temperature

## Greek symbols

$\Phi_{E}$, $\Phi_{C}$: Expressions for Cattaneo–Christov theory
$\underline{\underline{\tau }}$: Cauchy stress tensor
$$\sigma$$: Electrical conductivity
$$\mu_{f}$$: Dynamic viscosity
$\mu_{c}$: Cross-viscosity coefficient
$$\Omega$$: Angular velocity
$$\upsilon$$: Kinematic viscosity
$\lambda_{E}$: Heat flux relaxation time
$(\rho c)_{f}$: Fluid heat capacity
$(\rho c)_{p}$: Nanoparticles heat capacity
$\beta_{1} ,\beta_{2}$: Coefficients of heat and mass slip
$\theta$: Non-dimensional heat
$\varphi$: Non-dimensional nanoparticle volume fraction
$\gamma$: Thermal slip factor
$\lambda_{1}$: Energy diffusion Deborah number
$\lambda_{2}$: Mass diffusion Deborah number
$\alpha$: Rate of stretching parameter
$\delta$: Slip factor of nanoparticle volume fraction
