# Supplementary material for: Heat and mass flux through a Reiner–Rivlin nanofluid flow past a spinning stretching disc: Cattaneo–Christov model
Source: Sci Rep. 2022 Aug 24;12:14468. doi: 10.1038/s41598-022-18609-7 (PMC9402966; doi:10.1038/s41598-022-18609-7)
Supplement: Supplementary file 1 — Supplementary Information. [file 41598_2022_18609_MOESM1_ESM.pdf]

Appendix

The constants that appearing in Eqs. (48-51) may be listed as follows:

$c_1 = \dfrac{\alpha\, e^{-M\eta_\infty}}{2\cosh(M\eta_\infty)}$, $c_2 = \dfrac{\alpha\, e^{M\eta_\infty}}{2\cosh(M\eta_\infty)}$, $c_3 = \dfrac{S_w}{S_c} c_8 - \dfrac{1}{M}(c_1 - c_2)$, $c_4 = \dfrac{-\alpha\, e^{-M\eta_\infty}}{2\sinh(M\eta_\infty)}$, $c_5 = \dfrac{\alpha\, e^{M\eta_\infty}}{2\sinh(M\eta_\infty)}$

,

$c_6 = \dfrac{-1}{\gamma + \eta_\infty}$, $c_7 = \dfrac{\eta_\infty}{\gamma + \eta_\infty}$, $c_8 = \dfrac{-1}{\delta + \eta_\infty}$, $c_9 = \dfrac{\eta_\infty}{\delta + \eta_\infty}$.

The constants that appearing in Eqs. (52-55) may be listed as follows:

$c_{10} = c_1{}^2 - (1 - KM^2)(2c_1{}^2 + c_4{}^2)$,

$c_{11} = c_2{}^2 - (1 - KM^2)(2c_2{}^2 + c_5{}^2)$, $c_{12} = c_1 M(-2c_3 - K)$, $c_{13} = -c_2 M(-2c_3 - K)$,

$c_{14} = -2c_4c_5 + 4Kc_1c_2M^2 - 2Kc_4c_5M^2 + 6c_1c_2$, $c_{15} = \dfrac{c_{18} - c_{17}\, e^{-M\eta_\infty}}{2\sinh(M\eta_\infty)}$, $c_{16} = \dfrac{c_{17}\, e^{M\eta_\infty} - c_{18}}{2\sinh(M\eta_\infty)}$

$c_{17} = \dfrac{1}{M^2}(c_{14} - \dfrac{c_{10}}{3} - \dfrac{c_{11}}{3})$, $c_{18} = \dfrac{c_{14}}{M^2} - \dfrac{c_{10}}{3M^2} e^{2M\eta_\infty} - \dfrac{c_{11}}{3M^2} e^{-2M\eta_\infty} - \dfrac{c_{12}}{2M}\eta_\infty e^{M\eta_\infty} + \dfrac{c_{13}}{2M}\eta_\infty\, e^{-M\eta_\infty}$,

$c_{19} = \dfrac{S_w}{S_c}(c_{35} + c_{38}) - c_{20}$, $c_{20} = \dfrac{c_{10}}{6M^3} - \dfrac{c_{11}}{6M^3} - \dfrac{c_{12}}{2M^3} + \dfrac{c_{13}}{2M^3} + \dfrac{c_{15}}{M} - \dfrac{c_{16}}{M}$, $c_{21} = 2c_1c_4KM^2(1 - M)$,

$c_{22} = 2c_2c_5KM^2(1 + M)$, $c_{23} = -2c_3c_4M$, $c_{24} = 2c_3c_5M$

$c_{25} = c_1c_5(2KM^2 + 2KM^3 + 4) + c_2c_4(2KM^2 - 2KM^3 + 4)$ $c_{26} = \dfrac{c_{29} - c_{28}\, e^{-M\eta_\infty}}{2\sinh(M\eta_\infty)}$, $c_{27} = \dfrac{c_{28}\, e^{M\eta_\infty} - c_{29}}{2\sinh(M\eta_\infty)}$,

$c_{28} = \dfrac{1}{M^2}(c_{25} - \dfrac{c_{22}}{3}\dfrac{c_{21}}{3})$,

$c_{29} = \dfrac{c_{25}}{M^2} - \dfrac{c_{21}}{3M^2} e^{2M\eta_\infty} - \dfrac{c_{22}}{3M^2} e^{-2M\eta_\infty} - \dfrac{c_{22}}{2M}\eta_\infty e^{M\eta_\infty} + \dfrac{c_{24}}{2M}\eta_\infty\, e^{-M\eta_\infty}$,

$c_{30} = \dfrac{c_{34} + c_{32} - \gamma\, c_{33}}{\gamma + \eta_\infty}$, $c_{31} = c_{34} - c_{30}\eta_\infty$, $c_{32} = \dfrac{Prc_6}{M^3}[-2(c_1 - c_2) + \lambda_1(c_1{}^2 - c_2{}^2 + 4Mc_3(c_1 + c_2))]$,

$c_{33} = \dfrac{Prc_6}{M^2}[-2(c_1 + c_2) + \lambda_1(2c_1{}^2 + 2c_2{}^2 + 4Mc_3(c_1 - c_2))]$,

$c_{34} = -Pr[-2c_6(\dfrac{c_1}{M^3} e^{M\eta_\infty} - \dfrac{c_2}{M^3} e^{=M\eta_\infty} + \dfrac{c_3}{2}\eta_\infty{}^2) + 4\lambda_1 c_6(\dfrac{c_1{}^2}{4M^3} e^{2M\eta_\infty}$

$- \dfrac{c_2{}^2}{4M^3} e^{=2M\eta_\infty} + \dfrac{c_1c_3}{M^2} e^{M\eta_\infty} + \dfrac{c_2c_3}{M^2} e^{-M\eta_\infty}) - \dfrac{1}{2}(N_B c_6 c_8 + N_T c_6{}^2)\eta_\infty{}^2]$,

$c_{35} = \dfrac{c_{39} + c_{37} - \delta\, c_{38}}{1 + \eta_\infty}$, $c_{36} = c_{39} - \eta_\infty\, c_{35}$, $c_{37} = \dfrac{S_c c_8}{M^3}[-2(c_1 - c_2) + \lambda_2(c_1{}^2 - c_2{}^2 + 4Mc_3(c_1 + c_2))]$,

$c_{38} = \dfrac{S_c c_8}{M^3}[-2(c_1 + c_2) + \lambda_2(2(c_1{}^2 + c_2{}^2) + 4Mc_3(c_1 - c_2))]$, and

$c_{39} = -S_c c_8[-2(\dfrac{c_1}{M^3} e^{M\eta_\infty} - \dfrac{c_2}{M^3} e^{=M\eta_\infty} + \dfrac{c_3}{2}\eta_\infty{}^2) + \lambda_2(\dfrac{c_1{}^2}{M^3} e^{2M\eta_\infty}$

$- \dfrac{c_2{}^2}{M^3} e^{=2M\eta_\infty} + 4\dfrac{c_1c_3}{M^2} e^{M\eta_\infty} + 4\dfrac{c_2c_3}{M^2} e^{-M\eta_\infty})$.
